# Individual differences of conflict monitoring and feedback processing during reinforcement learning in a mock forensic context

**DOI:** 10.3758/s13415-020-00776-7

**Published:** 2020-02-10

**Authors:** Anja Leue, Katharina Nieden, Vera Scheuble, André Beauducel

**Affiliations:** 1 Institute of Psychology, University of Kiel, Olshausenstrasse 75, 24118 Kiel, Germany; 2 Institute of Psychology, University of Bonn, Bonn, Germany

**Keywords:** Conflict monitoring, Reinforcement learning, Personality, Reasoning, ERP

## Abstract

This study investigated individual differences of conflict monitoring (N2 component), feedback processing (feedback negativity component), and reinforcement learning in a discrimination learning task using a mock (fictitious) forensic scenario to set participants in a semantic task context. We investigated individual differences of anxiety-related, impulsivity-related traits and reasoning ability during trial-and-error learning of mock suspect and nonsuspect faces. Thereby, we asked how the differential investment of cognitive-motivational processes facilitates learning in a mock forensic context. As learning can be studied by means of time-on-task effects (i.e., variations of cognitive processes across task blocks), we investigated the differential investment of cognitive-motivational processes block-wise in *N* = 100 participants. By performing structural equation modeling, we demonstrate that conflict monitoring decreased across task blocks, whereas the percentage of correct responses increased across task blocks. Individuals with higher reasoning scores and higher impulsivity-related traits relied rather on feedback processing (i.e., external indicators) during reinforcement learning. Individuals with higher anxiety-related traits intensified their conflict monitoring throughout the task to learn successfully. Observation by relevant others intensified conflict monitoring more than nonobservation. Our data highlight that individual differences and social context modulate the intensity of information processing in a discrimination learning task using a mock forensic task scenario. We discuss our data with regard to recent cognitive-motivational approaches and in terms of reinforcement learning.

**Electronic supplementary material:**

The online version of this article (10.3758/s13415-020-00776-7) contains supplementary material, which is available to authorized users.

Since the initial publication of the (integrative) conflict monitoring theory (Botvinick, 2007; Botvinick, Braver, Barch, Carter, & Cohen, 2001; Botvinick, Cohen, & Carter, 2004) and its cognitive-motivational extensions (Botvinick & Braver, 2015; Braver et al., 2014), several studies have investigated the relationship of conflict monitoring and individual differences (Amodio, Master, Yee, & Taylor, 2008; De Pascalis, Varriale, & D’Antuono, 2010; Dennis & Chen, 2007; Leue, Lange, & Beauducel, 2012; Leue, Weber, & Beauducel, 2014). These studies were primarily based on go/no-go tasks asking participants to suppress a predominant reaction to no-go stimuli. In addition to the (integrative) conflict monitoring theory and models on cognitive control (Botvinick & Braver, 2015), reinforcement learning has been conceptually introduced by means of computational and hierarchical reinforcement learning models (Holroyd & Coles, 2002; Holroyd & Yeung, 2011, 2012; Umemoto, HajiHosseini, Yates, & Holroyd, 2017). Accordingly, some studies used learning tasks to investigate individual differences of conflict monitoring, feedback processing, and reinforcement learning (De Pascalis et al., 2010; Lange, Leue, & Beauducel, 2012; Scheuble, Nieden, Leue, & Beauducel,^2019^). However, systematic investigations of conflict monitoring and reinforcement learning in semantically embedded contexts are rare (but see Nieden, Scheuble, Beauducel, & Leue, 2020). Therefore, we used neuroscientific predictions and findings on individual differences of conflict monitoring and reinforcement learning to investigate the generalizability of these concepts to a forensic context.

## Conflict monitoring and reinforcement learning

To study individual differences of conflict monitoring and reinforcement learning (i.e., feedback-based learning), we refer to reviews (e.g., Botvinick, 2007; Botvinick & Braver, 2015; Holroyd & Coles, 2002; Holroyd & Yeung, 2012) and studies using simply structured experimental go/no-go tasks and discrimination learning tasks (Lange et al., 2012; Leue et al., 2012; Leue et al., 2014; Nieden et al., 2020; Scheuble et al., 2019). Botvinick (2007) argues that conflict-inducing stimuli function like “teaching signals” and are more cognitively demanding because conflict-inducing stimuli often require response adaptation (e.g., no-go stimuli require nonresponses in a series of go responses). When participants obtain feedback to learn the correct stimulus–response association, accounts on (hierarchical) reinforcement learning (Holroyd & Coles, 2002; Holroyd & Yeung, 2012) and mechanisms on motivation and cognitive control (Botvinick & Braver, 2015) are useful to explain the cognitive-motivational processes that are related to differential activations of the anterior cingulate cortex (ACC). Even when we did not investigate ACC functions directly, we investigate two event-related potentials (ERP) that have a neural generator in the ACC (Amodio et al., 2008; Leue et al., 2012; Nieuwenhuis, Yeung, van den Wildenberg, & Ridderinkhof, 2003) and that have been shown to reflect cognitive-motivational processes. These ERPs are the stimulus-locked N2 component and the feedback-locked FN component.

Van der Helden, Boksem, and Blom (2010) summarized learning “as the act, process, or experience of gaining knowledge or skill” (p. 1596). Our study starts with a situation of mock police officers learning to distinguish suspects and nonsuspects by means of feedback. Some learn under social observation, whereas others learn without social observation. We presume that reinforcement learning requires working memory resources (e.g., investment of cognitive demand) to perform the task successfully (Shenhav, Botvinick, & Cohen, 2013; Shenhav et al., 2017). Learning implies that people correctly differ between stimuli and responses or become familiar with predefined stimulus–response or stimulus–feedback associations (Holroyd & Coles, 2002; Holroyd, Pakzad-Vaezi, & Krigolson, 2008; Holroyd & Yeung, 2011, 2012). We presume that stimulus monitoring and response selection are cognitively demanding processes, resulting in a more negative N2 component, especially when stimulus–response differentiations have not yet been successfully learned (i.e., in the beginning of a learning task). When the stimulus–response differentiation of mock suspect and nonsuspect faces has been learned, the stimulus differentiation is less cognitively demanding, resulting in a less pronounced N2 component in later task blocks (Nieden et al., 2020). Moreover, more intense conflict monitoring should be related to faster learning of the correct stimulus classification. Accordingly, we presumed that more pronounced conflict monitoring (frontal N2 component) is related to a more pronounced learning slope (i.e., an increase of the percentage of correct responses with time on task; Hypothesis 1). When stimulus–response–feedback associations have been successfully learned, participants switch into performance monitoring. “Performance monitoring serves the correction, adaptation and optimization of actions” (Ullsperger, Danielmeier, & Jocham, 2014, p. 49). One opportunity to measure performance monitoring is given with feedback-related negativity (FRN, or FN; Gehring & Willoughby, 2002; Krigolson, 2018; Miltner, Braun, & Coles, 1997; Walsh & Anderson, 2012) and reward positivity (i.e., the feedback component that follows correct feedback and was earlier entitled as feedback correct-related positivity; Baker & Holroyd, 2011; Holroyd et al., 2008). Previous studies (Bellebaum & Daum, 2008; Bellebaum, Kobza, Thiele, & Daum, 2010) illustrated that a reduction of the FN (i.e., less negative amplitude) was related to having successfully learned in a guess task.

## Individual differences of conflict monitoring and reinforcement learning in a collaborative context: Previous findings

Nieden et al. (2020) investigated whether individual differences of conflict monitoring intensity (Botvinick, 2007) and reinforcement learning (Holroyd & Coles, 2002; Holroyd & Yeung, 2011) generalize to a context that asks participants to learn differentiating collaborative and noncollaborative faces. Noncollaborative faces elicited a more intense conflict monitoring than did collaborative faces. As time-on-task effects (i.e., variations of cognitive processes across task blocks) on behavioral and ERP data can be conceived as indicators of learning, we investigated variations of conflict monitoring intensity (N2 component) across three task blocks and feedback processing (FN component). We demonstrated that the frontal N2 component was most pronounced (i.e., more negative) in the initial task block compared with later task blocks. This finding suggests that participants invest more cognitive resources (e.g., degree of conflict monitoring) while learning the correct stimulus classification. In addition, they use feedback in later task blocks as a second loop of verification of their responses. With regard to individual differences, we observed a more pronounced frontal N2 amplitude following collaborative faces in individuals with higher versus lower trait-BIS scores in the initial compared with later task blocks. Observation intensified conflict monitoring more than nonobservation during learning. The discrimination learning task of Nieden et al. (2020) combined active learning by means of verbal feedback and observation versus nonobservation by relevant others (for reinforcement learning and observation, see Joiner, Piva, Turrin, & Chang, 2017). The manipulation of the observation factor was introduced because discrimination learning takes place under different observational conditions. In accordance with Nieden et al. (2020), we expected the N2 to be more pronounced under social observation versus nonobservation (Hypothesis 2).

## Conflict monitoring and reinforcement learning in a forensic context

For several years, psychophysiological investigations in the forensic context were mainly based on the Guilty Knowledge Test, or Concealed Information Test (CIT), and the related P3 amplitude difference between known, salient probe stimuli and unknown, less salient, irrelevant stimuli (Ben-Shakhar & Elaad, 2003; Leue & Beauducel, 2019; Meijer, Klein Selle, Elber, & Ben-Shakhar, 2014). Conflict monitoring and reinforcement learning have not yet been related to forensic experimental settings. However, the cognitive processes induced in CITs are more complex and are probably related to the investment and control of cognitive resources (Leue & Beauducel, 2019). Therefore, we adapted a discrimination learning task (which could also be named as a go/no-go learning task) that may be more suitable for the investigation of conflict monitoring and reinforcement learning in a forensic setting. One forensic setting that requires conflict monitoring and learning from feedback processing is the context of person identification.

Research on person identification reveals that correct person identification depends on contextual factors (e.g., lighting conditions, distance between observer and observed suspect) and individual differences (Noyes, Hill, & O’Toole, 2018; Sporer, Penrod, Read, & Cutler, 1995). Experimental factors of lineup presentations and cognitive determinants of neural processes such as stimulus monitoring, sensitivity to mismatch, and feedback processing (cf. Larson, Clayson, & Clawson, 2014) during person identification have not yet been studied (Valentine & Davis, 2015). Therefore, we investigate individual differences of the neural processes (e.g., conflict monitoring, feedback processing, learning) in a mock forensic context when mock police officers or lawyers differentiate information provided by witnesses or technical devices (e.g., cameras) about mock suspects and nonsuspects based on different modalities of information (e.g., verbal information, figural information, crime scene data).

## Individual differences of conflict monitoring and reinforcement learning: Previous findings

Leue et al. (2014) demonstrated that individual differences of reasoning ability (Burgess & Braver, 2010; Kyllonen & Christall, 1990; Süß, Oberauer, Wittmann, Wilhelm, & Schulze, 2002) are linked to N2-related variations of conflict-monitoring intensity. Individuals with higher reasoning ability revealed more intense conflict monitoring (i.e., more negative N2 component) in cognitively more demanding conditions of a go/no-go task. When individuals do not know the correct stimulus–response association, as in a discrimination learning task, the task is more difficult in the beginning. Thus, individuals with higher reasoning ability were thought to invest more conflict monitoring in the initial compared with the later task phase and, accordingly, to show a more pronounced learning slope for the correct differentiation of mock suspect and nonsuspect faces (Hypothesis 3).

Leue and Beauducel (2008) reported meta-analytic evidence for the relationship of behavioral performance parameters in reinforcement-related (learning) tasks with anxiety-related and impulsivity-related traits. This research is rooted in the revised reinforcement sensitivity theory (rRST; Corr, 2008; Gray & McNaughton, 2000). In rRST, a behavioral inhibition system (BIS) is differentiated from a behavioral approach system (BAS). Subsequently, we refer to trait-BIS as an anxiety-related trait linked to the BIS and to trait-BAS as an impulsivity-related trait associated with the BAS. Previous conflict monitoring studies showed that individuals with higher trait-BIS scores reveal a more negative N2 amplitude (e.g., Amodio et al., 2008; Leue et al., 2012). For a discrimination learning task, where the degree of conflict monitoring to be invested should decrease with time-on-task, we expected that higher trait-BIS individuals invest more conflict monitoring in the initial task block and show a more pronounced learning slope than would individuals with lower trait-BIS scores (Hypothesis 4). Additionally, in prior research on the FN component (De Pascalis et al., 2010; Lange et al., 2012), we observed that individuals with higher versus lower trait-BIS scores learn more intensely from negative feedback. In contrast, more reward-sensitive individuals (e.g., higher scores in trait-BAS or extraversion) compared with fewer reward-sensitive individuals learn more intensely from positive feedback (e.g., Lange et al., 2012; Smillie, Cooper, & Pickering, 2011). Accordingly, we expect that a more pronounced (i.e., more positive) correct FN component predicts a more pronounced learning slope of higher versus lower trait-BAS individuals (Hypothesis 5). Relatedly, Scheuble et al. (2019) provided evidence on trait-BAS differences and interactions of reasoning ability on N2-related monitoring processes and reinforcement learning in a digit task, especially when the context comprised positively motivating cue words.

## Aims and hypotheses

**Fig. 1:**
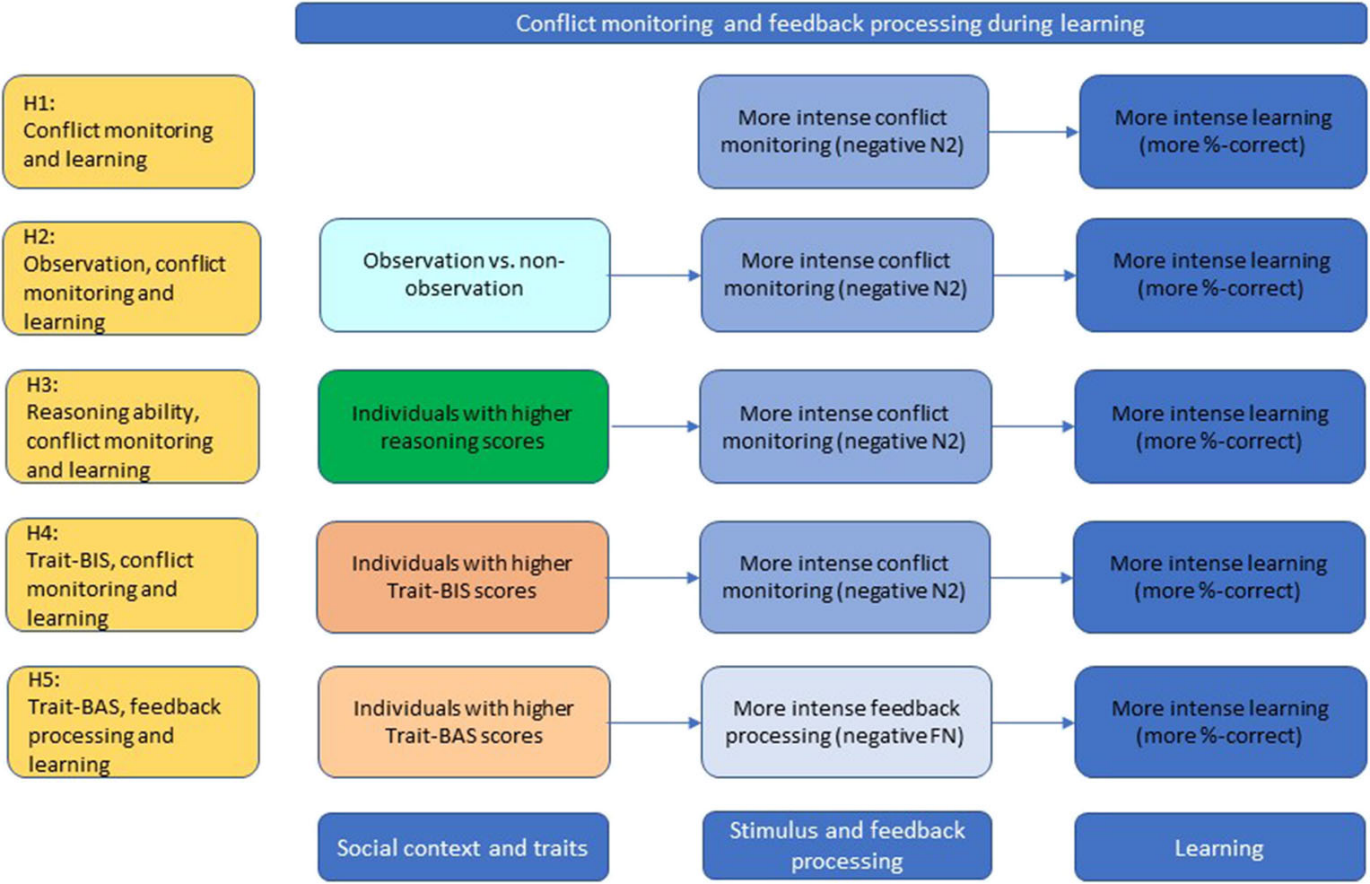
Summary of hypotheses

In sum, we investigated hypotheses based on the conflict monitoring account, the reinforcement learning theory, and the rRST in a discrimination learning task adapted for a forensic context. Accordingly, we expected that more intense conflict monitoring (frontal N2 component) is associated with a more pronounced learning slope (i.e., a more pronounced increase of the percentage of correct responses across time-on-task; Hypothesis 1). We predicted that observation in a learning task intensifies conflict monitoring (i.e., more negative N2 component) and learning compared with nonobservation (Hypothesis 2). Individuals with higher reasoning scores invest more conflict monitoring in the initial task block and show a more pronounced learning slope for the correct differentiation of mock suspect and nonsuspect faces across all task blocks (Hypothesis 3). Individuals with higher trait-BIS scores anticipate negative consequences of erroneous face classifications. Therefore, they invest more conflict monitoring in the initial block and show a more pronounced learning slope than individuals with lower trait-BIS scores (Hypothesis 4). Individuals with higher trait-BAS scores learn from positive feedback. That is why, we presumed that a more pronounced FN component (i.e., more intense surprise of positive feedback) predicts a more pronounced (i.e., more positively motivated) learning slope (i.e., higher percentage of correct responses) of higher versus lower trait-BAS individuals (Hypothesis 5). Hypotheses 1–5 are summarized in Fig. 1.

## Method

### Participants

A total of *N* = 130 subjects participated voluntarily in this study (another paradigm on conflict monitoring for the differentiation of collaborative versus noncollaborative faces is described in Nieden et al., 2020). Almost all (*n* = 119) participants were students, *n* = 6 participants were employees, *n* = 4 participants were unemployed on the date of examination, and *n* = 1 participant was a freelancer. All participants gave written informed consent at the beginning of the study. All participants were right-handed and had normal or corrected-to-normal vision.

In order to elucidate effects of learning as predicted in our hypotheses, we analyzed time-on-task effects of the ERP and behavioral data. Therefore, we divided the discrimination learning task into three blocks of equal length. Accordingly, we focused our analyses on participants with a sufficient number of artifact-free N2, FN, and behavioral data in each block. For block-wise analyses of the behavioral data, N2 and FN data, a subsample of the same *N* = 100 (30 male) participants was available (age: *M* = 24.06 years, *SD* = 3.78, range: 18–41 years) who met for the FN component the lower bound criterion of on average at least 10 epochs across picture type per block (Marco-Pallares, Cucurelli, Münte, Strien, & Rodriguez-Fornells, 2011). The descriptives of the artifact-free epochs for the block-wise N2 and FN components are summarized in Table 1.

**Table 1:** Means and standard deviations (in parentheses) of the number of artifact-free epochs for the N2 component and FN component (*N* = 100)

	Mock suspect faces	Mock nonsuspect faces	*t* test for dependent samples
*N2 component*			
Block 1	43.62 (7.99)	37.09 (8.97)	*t*(99) = 7.80; *p* = .99
Block 2	47.52 (8.91)	45.93 (8.73)	t(99) = 3.44; *p* = .99
Block 3	48.62 (8.73)	48.16 (8.30)	t(99) = 0.55; *p* = .70
*FN component*			
Block 1	25.36 (12.07)	23.10 (10.81)	*t*(99) = 2.09; *p* = .98
Block 2	29.68 (12.74)	30.51 (12.07)	t(99) = −0.69; *p* = .25
Block 3	30.16 (12.31)	32.30 (11.80)	t(99) = −1.81; *p* = .04

*Note.* Artifact-free epochs are reported for correct responses of the respective ERP component

### Measures

Participants completed the German version of Carver and White’s BIS/BAS scales (Strobel, Beauducel, Debener, & Brocke, 2001). We have chosen the BIS/BAS scales although meanwhile further valuable questionnaires for the assessment of trait-BIS and trait-BAS have been published (Pugnaghi, Cooper, Ettinger, & Corr, 2017; Reuter, Cooper, Smillie, Markett, & Montag, 2015). As these questionnaires had not yet been available in a published German translation when we planned the project, we applied the BIS/BAS scales in the present study. Moreover, several previous studies on conflict monitoring and trait-BIS used the BIS/BAS scales (e.g., Amodio et al., 2008; Leue et al., 2012) so that the use of these scales enhances the comparability of results with previous studies. The BIS scale assesses sensitivity to aversiveness and comprises seven items (Cronbach’s alpha: .83 for the block-wise sample of *N* = 100). The total BAS scale assesses sensitivity to appetitive reinforcement and comprises 13 items (Cronbach’s alpha: .71 for the block-wise sample of *N* = 100). Handedness was measured with the German version of the Edinburgh Handedness Inventory comprising 10 items (Milenkovic & Dragowic, 2013; Oldfield, 1971).

To assess participants’ reasoning ability, we applied three subtests of the Intelligence-Structure Test 2000 R (I-S-T 2000 R; Beauducel, Liepmann, Horn, & Brocke, 2010; Liepmann, Beauducel, Brocke, & Amthauer, 2007). Subtest 2, Analogies, assessed verbal reasoning abilities. Participants chose one out of four presented words that best represented the relationship to a given word (e.g., *forest* : *trees* = *meadow* : (a) *grass* (b) *hay* (c) *food* (d) *green* (e) *grazing land*). Subtest 4, Arithmetics, assessed numerical reasoning abilities. Participants were asked to solve a number of arithmetic problems (e.g., 60 − 10 = A; A = ?). Subtest 9, Matrices, assessed figural reasoning ability. Participants were asked to choose one out of four figures that should complete a given figure sequence. Time to report solutions per subtest was limited: Participants had 7 minutes to complete Subtest 2, 10 minutes to perform Subtest 4, and 10 minutes for Subtest 9. Cronbach’s alpha collapsed across the three subtests of the basic module, Form A was .75 for *N* = 100 participants.

### Discrimination learning task

The discrimination learning task consisted of 16 face pictures from the Radboud Faces Database (Langner et al., 2010). We selected eight faces of neutral affect that were predefined to represent mock suspects (four male: Rafd090_03, Rafd090_20, Rafd_090_23, Rafd090_25; four female: Rafd090_16, Rafd090_18, Rafd090_31, Rafd090_37). Another subset of eight faces of neutral affect were predefined to represent mock nonsuspects (four male: Rafd090_28, Rafd090_36, Rafd090_38, Rafd090_71; four female: Rafd090_56, Rafd090_57, Rafd090_58, Rafd090_61). Intensity (*M*_sus_ = 3.40, *SD*_sus_ = 0.39, range_sus_: 2.82–4.09 vs. *M*_nonsus_ = 3.55, *SD*_nonsus_ = 0.20, range_nonsus_: 3.36–3.92), realness (*M*_sus_ = 3.91, *SD*_sus_ = 0.32, range_sus_: 3.38–4.26 vs. *M*_nonsus_ = 4.15, *SD*_nonsus_ = 0.12, range_nonsus_: 3.92–4.35) and valence (*M*_sus_ = 3.14, *SD*_sus_ = 0.34, range_sus_: 2.52–3.46 vs. *M*_nonsus_ = 3.17, *SD*_nonsus_ = 0.23, range_nonsus_: 2.81–3.50) were similar for face stimuli of mock suspects versus nonsuspects (Langner et al., 2010).

The sequence of faces of mock suspects and nonsuspects was pseudorandomized, so the same sequence of pictures was presented to each participant. Participants were instructed to learn which face indicated a mock suspect and which face indicated a mock nonsuspect. They were asked to respond to a mock suspect face by pressing the space bar after face presentation and to withhold responses when a mock nonsuspect face was presented. Thus, faces that indicated a mock suspect required a go response, and faces that showed a mock nonsuspect required a no-go (i.e., withholding) response. We instructed go responses to mock suspect faces and no-go responses to mock nonsuspect faces, as this stimulus–response association is compatible with lineup presentations in person identification settings (Valentine & Davis, 2015). Each of the eight mock suspect faces and each of the eight mock nonsuspect faces were presented 21 times, resulting in a total of 168 trials, comprising mock suspect faces that required a go response and 168 trials of mock nonsuspect faces that required withholding a response. We have applied an equal number of go and no-go stimuli because participants performed a learning task. If one stimulus type had a lower probability than the other stimulus type, learning might be facilitated to the more frequently presented stimulus type. Therefore, a 50:50 ratio of go and no-go stimuli keeps the option to test whether the neural processes are of differential or equal intensity to go (mock suspect faces) versus no-go (mock nonsuspect faces) stimuli. Previous go/no-go tasks also applied go and no-go stimuli equiprobably (Huster, Enriquez-Geppert, Lavallee, Falkenstein, & Herrmann, 2013; Larson et al., 2014). However, these articles did not investigate learning tasks with go versus no-go stimuli. All instructions were presented on a 17-in. TFT screen. The face pictures were 340 pixels wide × 512 pixels high (2.88 cm wide × 4.33 cm high). All faces were centrally presented on the screen.

Before the task, participants listened to the description of a crime vignette via STIM earphones. The vignette is given in Supplement S1. This crime vignette was applied before task performance to place participants in the role of the mock police officers working on the murder case to disentangle mock suspects and nonsuspects. No further information about mock suspects versus nonsuspects was given in the task description. Thus, participants were asked to learn, by trial-and-error responses, which of the eight faces represented a predefined mock suspect, and which of the other eight faces represented no predefined mock suspect.

**Fig. 2:**
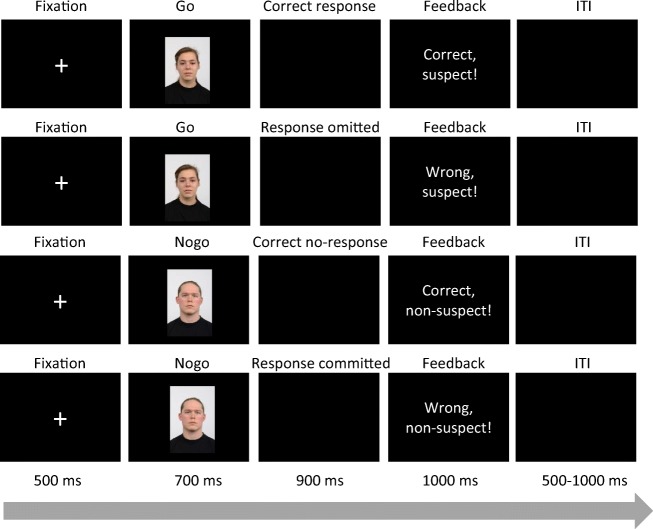
Trial sequence for mock suspect faces (go stimulus) and mock nonsuspect faces (no-go stimulus) and corresponding responses.

Each trial started with a fixation cross that was presented in the center of the screen for 500 ms. Each face was presented for 700 ms. When the picture disappeared, participants could respond within 900 ms to indicate whether a face represented a mock suspect. The screen remained black during the response interval. After the response interval, a feedback was presented on the screen for 1,000 ms, depending on the picture type and the reaction. Similar to the postidentification feedback studied in a previous meta-analysis (Douglass & Steblay, 2006), and in one of our previous reinforcement learning studies (Nieden et al., 2020), we provided the following trial-by-trial feedback: Correct reactions to mock suspect faces resulted in a positive feedback (“Right, suspect”). The same was true for nonresponses to mock nonsuspect faces (“Right, no suspect”). Withholding reactions to mock suspect faces were associated with a negative feedback (“Wrong, suspect”). Erroneous responses to mock nonsuspect faces also resulted in negative feedback (“Wrong, no suspect”). The intertrial interval (ITI) varied between 500 and 1,000 ms. Performing the task took approximately 20 minutes. Figure 2 gives an example of the four trial sequences that occurred during the task.

### Procedure

Participants were recruited through Facebook student groups and announcements on bulletin boards. The announcements included the following selection criteria: Participants should be right-handed. They should not suffer from neurological diseases (e.g., epilepsy) and be between 18 and 45 years of age. Everyone who was interested in taking part in this study was asked to choose his or her favored examination date in a doodle questionnaire. Participants received an e-mail including the date of the examination and further important information concerning the examination (e.g., sleeping as usual the day before participation, avoiding substance use of coffee, black tea, and medication the day before of the study).

Each participant was asked to bring a related person the day of the examination. (A related person was defined as a good friend, a family member, or a fellow student.) This procedure was chosen to ensure that half of the sample could be included in the observation condition. All participants received a reminder e-mail the day before the examination to minimize unexpected cancellations.

After arriving, participants gave written informed consent in accordance with the revised 2013 Helsinki Declaration. The experimenter gave all important information regarding duration of the examination, questionnaires, and instructions of the experimental task. Participants were seated in a comfortable chair, approximately 80-cm from the screen. They were told to choose a comfortable seating position to easily tap the space bar with their right index finger. A subsample of *n* = 50 of the *N* = 100 participants brought a related person with them. The related person was seated in an adjacent room that was connected to the experiment room through a one-way window. The related person observed the participant during task performance. The participant was told that he or she would be observed during task performance.

Presentation V16.5 (Neurobehavioral Systems, Albany, NY, USA) was used to present the experimental task (all instructions were given in white [255, 255, 255], 30-pt Arial font on a black screen). The experimental task started with 10 examination trials to ensure that the participants had understood the instructions correctly. Participants were explicitly requested to ask questions if something remained unclear. By tapping the space bar, participants started the main part of the task. The EEG was recorded during task performance. After performing the experimental task, participants could wash their hair. The observer was paid and dismissed (€8.50). The participant who had performed the task changed the room and completed the I-S-T 2000 R and the BIS/BAS scales. Each examination lasted about 3 hours. At the end of the examination, participants were thanked, dismissed, and paid (€25.50).

### EEG recording and processing

**Fig. 3:**
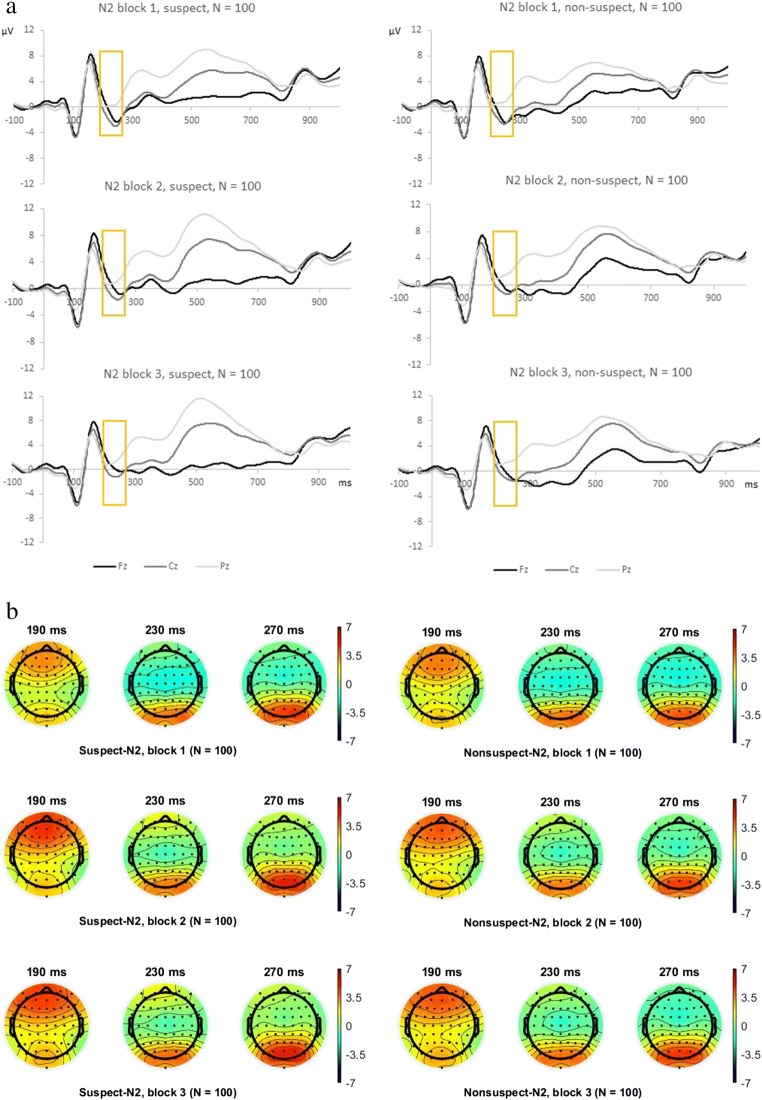
**a** Grand averages illustrating the stimulus-locked N2 component with correct responses for (**a**) mock suspect faces in Task Blocks 1–3 and for (**b**) mock nonsuspect faces for Task Blocks 1–3 at Fz, Cz, and Pz (*N* = 100). **b** Topographical plots for mock suspect and nonsuspect faces with correct responses of the N2 time range in Task Blocks 1–3. Legend is given in microvolt

The EEG was recorded using the ActiveTwo EEG system (BioSemi, Amsterdam, Netherlands) with 64 scalp active electrodes. Two electrodes were placed beyond the epicanthi of both eyes to record the horizontal electrooculogram (HEOG). Additionally, one electrode was placed approximately 1 cm below the right eye to measure the vertical electrooculogram (VEOG). ActiView software V7.06 (BioSemi) was used to digitize all bioelectric signals. The EEG was sampled at 512 Hz. Electrode offsets were kept below 30 mV during EEG recording. Off-line analysis was performed by using EEGLAB V13.4.4 (Delorme & Makeig, 2004) based on MATLAB R2015 (The MathWorks). EEG data were off-line band-pass filtered (1–15 Hz; cf. Leue, Klein, Lange, & Beauducel, 2013; Widmann, Schröger, & Maess, 2015) and re-referenced to averaged P9/P10 electrodes (as in our previous EEG studies), which are near to the mastoids and probably capture less muscle noise from the neck (Luck, 2014). One epoch incorporated a 100-ms baseline and lasted until 1,600 ms after stimulus onset (we present an epoch length of −100 to 1,000 ms poststimulus). An independent component analysis (ICA; automated infomax decomposition with adjust algorithm; Mognon, Jovicich, Bruzzone, & Buiatti, 2011) was applied to correct for ocular artifacts. Further technical and muscle artifacts were rejected when the EEG signal exceeded ±85 μV (cf. Leue et al., 2012; Leue et al., 2014). The EEG data were segmented for correct reactions to mock suspect and nonsuspect faces. The block-wise grand averages indicate (see Fig. 3a) that the N2 component occurred for mock suspect and nonsuspect faces between 190 and 270 ms after stimulus onset. The N2 component for correct reactions was quantified separately for each participant as a mean amplitude in the 190–270 ms interval (Luck, 2014). In addition to the N2 amplitude, we quantified the FN. EEG-data were off-line bandpass-filtered (0.1–20 Hz, referring to prior FN research; e.g., Leue, Cano Rodilla, & Beauducel, 2015) and re-referenced to averaged mastoids (P9/P10). One epoch incorporated a 100-ms baseline and lasted until 1,000 ms after feedback onset. An ICA was applied to correct for ocular artifacts. Further technical and muscle artifacts were rejected when the EEG signal exceeded ±85 μV.

**Fig. 4:**
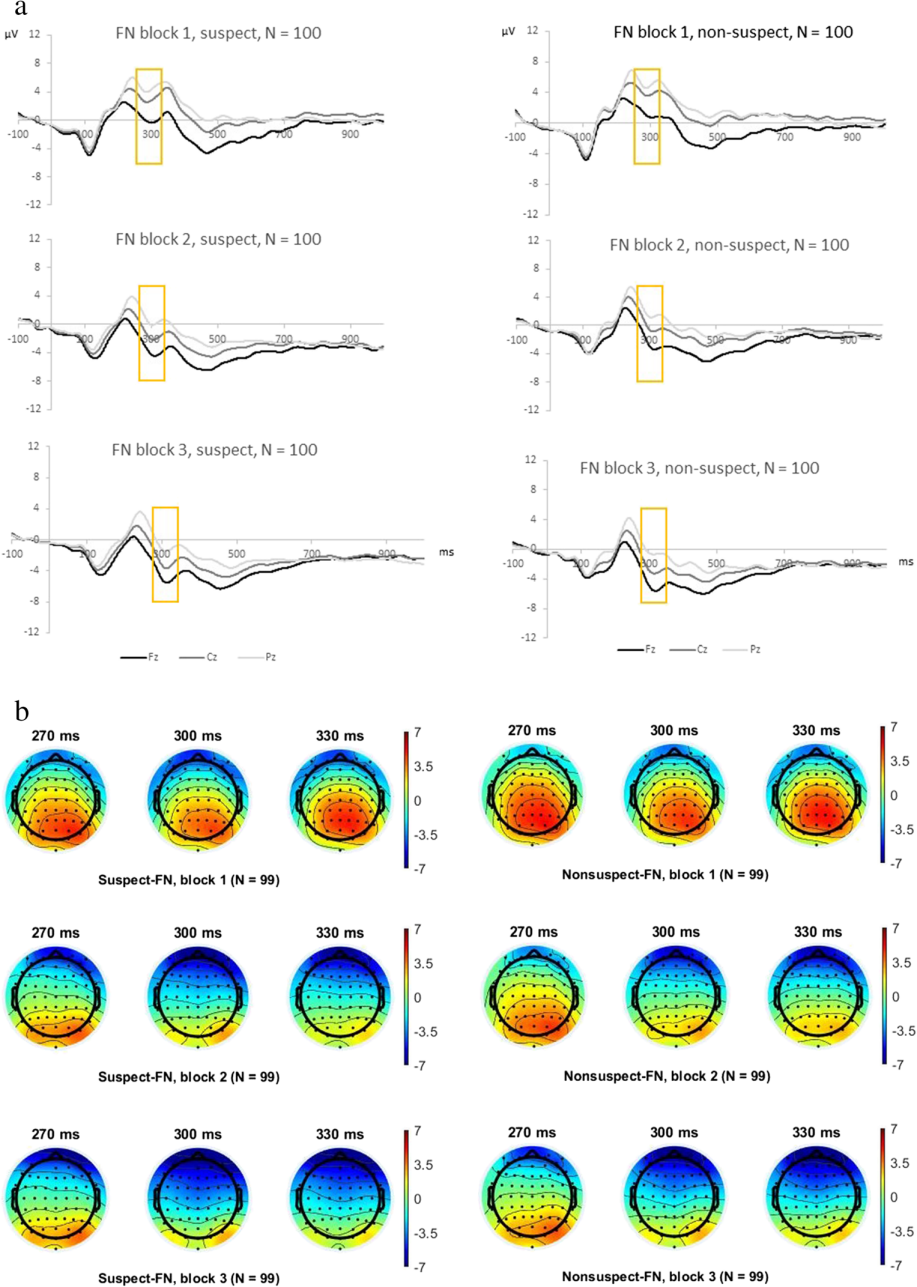
**a** Grand averages illustrating the feedback-locked FN component with correct responses for (**a**) mock suspect faces in Task Blocks 1–3 and for (**b**) mock nonsuspect faces for Task Blocks 1–3 at Fz, Cz, and Pz (*N* = 100). **b** Topographical plots for mock suspect and nonsuspect faces with correct responses of the N2 time range in Task Blocks 1–3. Legend is given in microvolt

For block-wise analyses of the N2 and FN, each of the three task blocks consisted of 112 successive trials. Each block exclusively included correct reactions to mock suspect and nonsuspect faces (see Figs. 3a and 4a). The FN epochs were segmented for correct reactions to mock suspect faces and correct reactions for mock nonsuspect faces (correct feedback) as well as for erroneous reactions to mock suspect and nonsuspect faces (not analyzed here). The mean FN amplitude following correct feedback was quantified across the three task blocks between 270 and 330 ms (see Fig. 4a) and separately for each participant. As the N2 and the FN component could be clearly detected in the grand average (as in our previous contextualized go/no-go task; Nieden et al., 2020), we quantified both components as mean amplitudes (Luck, 2014) instead of performing a principal component analysis as in Scheuble et al. (2019).

### Statistical analysis

We investigated a structural equation model for frontal electrodes (collapsed across F3, Fz, F4) and central electrodes (collapsed across C3, Cz, C4; see Supplement S2) because the N2 component and the FN component typically have a frontocentral topography (Amodio et al., 2008; Lange et al., 2012; Leue et al., 2012). We performed structural equation modeling in order to calculate latent variables for trait-BIS, trait-BAS, and reasoning ability in the context of a growth model representing the time-on-task effects on the N2 and the FN. The statistical power for the detection of effects has been shown to be superior for latent variable modeling when compared with models that are based on sum scales (Scheuble et al., 2019). We specified one model including block-wise data of the frontal mean N2 amplitude (collapsed across F3, Fz, F4), the frontal mean FN amplitude (collapsed across F3, Fz, F4), and the percentage of correct responses as dependent variables using Mplus (Muthén & Muthén, 1998–2017, Version 8). We entered trait-BIS, trait-BAS, and reasoning ability as latent variables with unit variance that were based on parcels. Parceling measurement variables allows for a more robust estimation of latent variables than single items do because parceling helps to eliminate theoretically unimportant noise (Matsunga, 2008, p. 289). Each parcel incorporated three to five items resulting in two parcels for trait-BIS, three parcels for trait-BAS, and four parcels for verbal, figural, and numerical reasoning. Each item parcel revealed a corrected item-total correlation of ≥.10 (leading to exclusion of Parcel 4 for verbal, numerical, and figural reasoning). To test Hypotheses 1–5 on individual differences of conflict monitoring, feedback processing and learning (i.e., percentage of correct responses) within one model, we computed a structural equation model comprising a latent intercept variable and a latent slope variable for the N2 amplitude containing the frontal N2 amplitude for mock suspect faces and the frontal N2 amplitude for mock nonsuspect faces in each of the three task blocks as measured variables. Similarly, a latent intercept and slope variable of the frontal FN amplitude was performed containing the frontal FN amplitude for mock suspect faces and the frontal FN amplitude for mock nonsuspect faces in each of the three task blocks as measured variables. Thus, in the growth model intercept parameters were estimated in order to represent the overall magnitude of the dependent variables (percentage correct responses, N2 amplitude, and FN amplitude), whereas the slope parameter represents the change of these dependent variables across the three task blocks. We defined predictions of the three latent intercept and slope variables on the latent trait variables (trait-BIS, trait-BAS, reasoning) and on gender (female vs. male) as well as observation (yes vs. no). The residuals of the following measured variables were allowed to correlate to fit the model: frontal N2 amplitude for mock suspect faces in Block 3 and Block 2, frontal N2 amplitude for mock suspect faces in Block 1 and Block 2. Verbal reasoning (Parcel 1) with verbal reasoning (Parcel 3), figural reasoning (Parcel 1) with figural reasoning (Parcel 2). The residuals of the percentage of hits were allowed to correlate in Blocks 2 and 3 for mock suspect faces and in Blocks 1 and 2 for mock nonsuspect faces.

The test for the multivariate normal distribution of the measured variables (Prelis 2.80; Jöreskog & Sörbom, 1999) was significant (χ^2^ = 122.13, *df* = 2, *p* < .001). Therefore, we used the Satorra–Bentler scaled robust maximum likelihood estimator (MLM) in order to compensate for a nonnormal multivariate distribution of the measured variables. For all analyses we report standardized path coefficients (β). We report the root mean square error of approximation (RMSEA), the standardized root mean residual (SRMR), and the comparative fit index (CFI) as model fit indices with suggested cutoff values (Beauducel & Wittmann, 2005; Raykov, 1998), indicating a good model fit (RMSEA ≤ .08, SRMR ≤ .08, CFI > .90) along with a chi-square (χ^2^) test of model fit. We applied SPSS 24 for paired *t* tests of picture classifications (see manipulation check) and integration of N2, FN, performance data, and trait variables into one file prior to Mplus analyses.

## Results

### Manipulation check

Participants were asked to evaluate the pictures as mock suspect versus nonsuspect faces (1 = correct classification, 0 = incorrect classification) after performing the task to see whether they had correctly learned the picture classification (eight mock suspect faces vs. eight mock nonsuspect faces). For statistical analysis, we included all participants who had completely evaluated the pictures without missing values. The *t* test for paired samples revealed that the correct classification of mock suspect and nonsuspect faces, respectively, did not substantially differ, *t*(94) = −0.69, *p* = .49. The sum scores were highly similar for the correct classifications of mock suspect faces (*M* = 7.87, *SD* = 0.36) and mock nonsuspect faces (*M* = 7.90, *SD* = 0.41), and very close to the maximum of eight correct mock suspect and eight correct mock nonsuspect classifications.

**Fig. 5:**
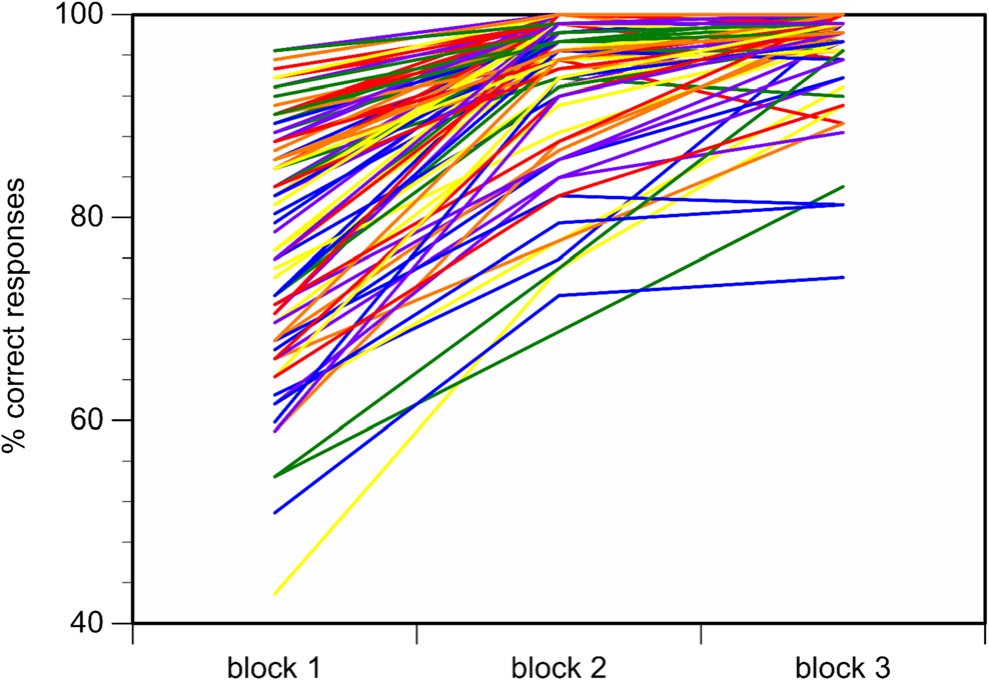
Learning curves for the percentage of correct responses for *N* = 100 participants in the three task blocks

To illustrate the learning effect in the present task, we calculated the percentage of correct responses to mock suspect and nonsuspect faces for each of the three task blocks as follows: (100% / 112 successive trials per block) × number of correct responses per block. The repeated-measures ANOVA including the percentage of correct responses per task block revealed a significant task block effect, *F*(2, 198) = 298.33, *p* < .01, ε = .64, η_p_^2^ = .75, with an increasing percentage of correct responses from Block 1 (*M* = 78.95%, *SE* = 1.20) to Block 2 (*M* = 93.94%, *SE* = 0.73), *F*(1, 99) = 408.46, *p* < .01, *η*_*p*_^*2*^ = .81, and from Block 2 to Block 3 (*M* = 97.05%, *SE* = 0.48), *F*(1, 99) = 36.72, *p* < .01, η_p_^2^ = .27 (for individual learning curves, see Fig. 5).

### Results of the frontal N2 amplitude, frontal FN amplitude, and percentage of correct responses

**Fig. 6:**
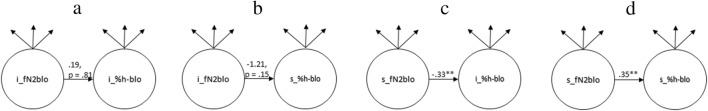
Part 1 of the structural equation model, with a completely standardized solution including the intercept or the slope of the frontal mean N2 amplitude across all three blocks, and the intercept or the slope of the percentage of correct responses across all three task blocks (Hypothesis 1). **a** Prediction of the %hit intercept (i_%h-blo) by the intercept of the N2 (i_fN2blo). **b** Prediction of the %hit slope (s_%h-blo) by the intercept of the N2 (i_fN2blo). **c** Prediction of the %hit intercept (i_%h-blo) by the slope of the N2 (s_fN2blo). **d** Prediction of the %hit slope (s_%h-blo) by the slope of the N2 (i_fN2blo). *p < .05. **p < .01. All *p*s are reported two-tailed

**Fig. 7:**
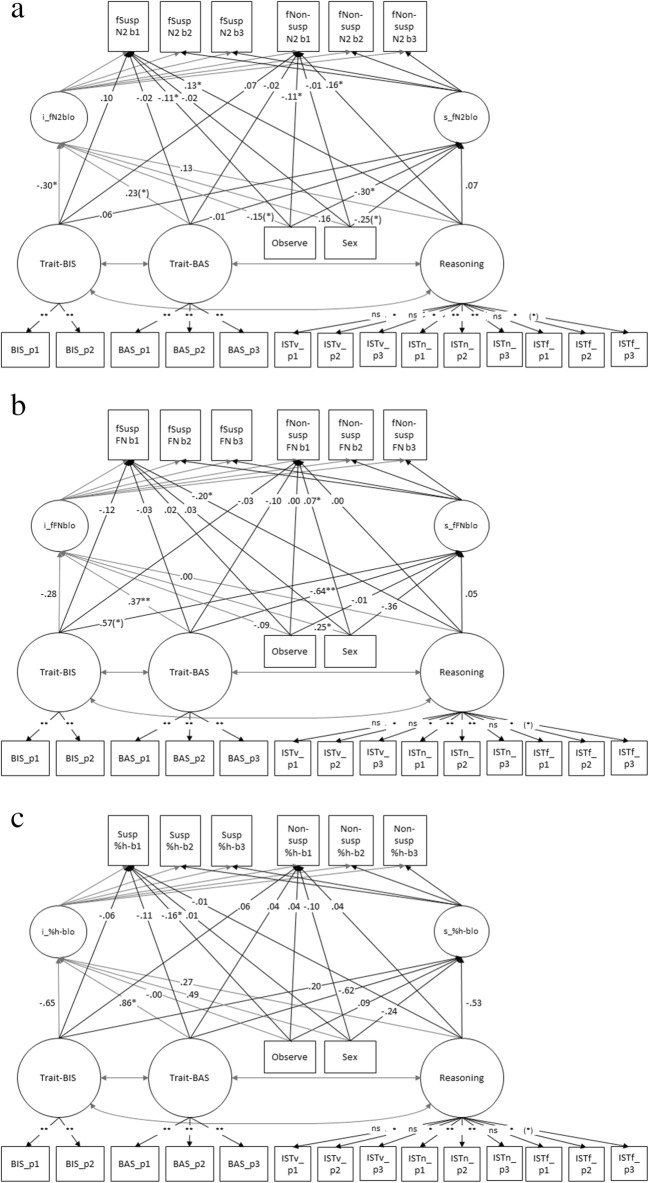
**a** Part 2 of the structural equation model, with a completely standardized solution including the intercept and the slope of the frontal N2 amplitude in Blocks 1, 2, and 3 for mock suspect and nonsuspect faces (inserted as measurement variables), with trait-BIS, trait-BAS, and reasoning regressed on the intercept and slopes. **b** Part 3 of the structural equation model, with a completely standardized solution including the intercept and the slope of the frontal FN amplitude in Blocks 1, 2, and 3 for mock suspect and nonsuspect faces (inserted as measurement variables), with trait-BIS, trait-BAS, and reasoning regressed on the intercept and slopes. **c** Part 4 of the structural equation model, with a completely standardized solution including the intercept and the slope of the percentage of correct responses in Blocks 1, 2, and 3 for mock suspect and nonsuspect faces (inserted as measurement variables), with trait-BIS, trait-BAS, and reasoning regressed on the intercept and slopes. Trait-BIS and trait-BAS correlated *r* = −.02, *p* = .86. Trait-BIS and reasoning correlated *r* = −.38, *p* < .01. Trait-BAS and reasoning correlated *r* = .20, *p* = .07. Trait-BIS correlated with sex *r* = .62, *p* < .01 and with observation *r* = −.11, *p* = .25. Trait-BAS correlated with sex *r* = −.02, *p* = .90, and with observation *r* = −.09, *p* = .30. Reasoning correlated with sex *r* = −.31, *p* < .01, with observation *r* = .08, *p* = .43. In **a, b,** and **c** we inserted observation (1 = yes, 0 = no) and sex (1 = male, 2 = female) as measurement variables. Trait-BIS, trait-BAS, and reasoning were computed as latent variables based on item parcels. In all figures, we present path coefficients as β *(N* = 100). Correlation coefficients are presented between item parcels and latent trait-variables. *p < .05. **p < .01. (*)*p* < .10. All *p*s are reported two-tailed

The model fit criteria suggested an acceptable overall fit (χ^2^ = 644.41, *df* = 463, *p* < .001, RMSEA = .06, SRMR = .10, CFI = .92). The overall growth model comprises three sets of parameter estimates that are relevant for testing Hypotheses 2–5: (1) beta weights between the latent traits (trait-BIS, trait-BAS, and reasoning) or the group variable (observation or sex) and the slope of the latent dependent variable; (2) beta weights between the latent traits (trait-BIS, trait-BAS, and reasoning) or the group variable (observation or sex) and the intercept of the latent dependent variable; and (3) beta weights between the latent traits (trait-BIS, trait-BAS, and reasoning) or the group variable (observation or sex) and the dependent measurement variable in Block 1. In addition to the overall growth model, we performed two separate growth models comprising either mock suspect faces or mock nonsuspect faces with all the other variables being equal to the overall model. We describe all results for Hypotheses 2–5 for slope, intercept and measurement level of the overall growth model followed by the results of the separate growth models for mock suspect and nonsuspect faces, respectively. For a complete overview and summary of the model findings, see Figs. 6 and 7. A summary of the separate analyses for mock suspect and nonsuspect faces is given with the figures in Supplements S4 and S5.

### Hypothesis 1: A more negative N2 component is associated with a more pronounced learning slope

Hypothesis 1 presuming that a more pronounced frontal N2 amplitude predicted an increase in percentage of the correct responses (briefly: %-correct responses) across all task blocks was confirmed. When the frontal N2 amplitude became more pronounced (i.e., more negative) across task blocks, the overall percentage of correct responses was higher (β_slope vs. intercept_ = −0.33, *p* < .01; see Fig. 6c). In addition, when conflict monitoring was most intense (i.e., most negative N2 amplitude) in the first task block and became less intense in the later blocks (slope N2), the percentage of correct responses increased with time-on-task (β_slope vs. slope_ = 0.35, *p* < .01; see Fig. 6d). As FN findings and learning were not part of Hypothesis 1, we present these findings in Supplement S3.

### Hypothesis 2: We predicted that observation in a learning task intensifies conflict monitoring (i.e., more negative N2 component) and learning compared with nonobservation

Hypothesis 2 was confirmed. As indicated by Fig. 7a for the slope effect of the frontal N2 amplitude, observation by relevant others intensified conflict monitoring across task blocks (β_slope_ = −.30, *p* < .05). Observation intensified the conflict monitoring to mock nonsuspect faces in Block 1 (β_b*1*_ = −.11, *p* < .05) and to mock suspect faces in Block 1 (β_b1_ = −.11, *p* < .05). Effects of observation were not found for the percentage of correct responses (β_intercept_ = −.00, β_slope_ = .09; see Fig. 7c). However, observation increased the percentage of correct responses to suspect faces even in Block 1 (see Fig. 7c, β_b1_ = −.16, *p* < .05), but not to nonsuspect faces (see Fig. 7c, β_b1_ = .04). When the models were run separately for mock suspect and nonsuspect faces, the observation effect was significant exclusively to mock suspect faces for the frontal N2 amplitude in Block 1 (β_suspect,b1_ = −.18, *p* < .05) and for the percentage of correct responses in Block 1 (β_suspect,b1_ = −.17, *p* < .05), but not to mock nonsuspect faces in Block 1 (see Supplement S4a for mock suspect faces, and Supplement S5 for mock nonsuspect faces).

### Hypothesis 3: Individuals with higher reasoning scores invest more conflict monitoring in the initial task block and therefore show a more pronounced learning slope for the correct differentiation of suspect and nonsuspect faces across task blocks

Contrary to Hypothesis 3, we did not observe evidence (neither for the intercept of the frontal N2 nor for the slope of the frontal N2) that individuals with higher reasoning scores invested more conflict monitoring within the task (β_intercept_ = .13) or across task block (β_slope_ = .07; see Fig. 7a). Individuals with higher reasoning scores revealed a significantly less negative frontal N2 amplitude in Block 1 for mock nonsuspect faces (β_nonsuspect, b1_ = .16, *p* < .05; see Fig. 7a) and a significantly less negative frontal N2 amplitude in Block 1 to mock suspect faces (β_b1_ = .13, *p* < .05; see Fig. 7a). In separate analyses of mock suspect versus nonsuspect faces, the reasoning effect of the frontal N2 in Block 1 was no longer significant to mock suspect faces (β_suspect, b1_ = .12, *p* = .22; see Supplement S4a) and to nonsuspect faces (β_nonsuspect, b1_ = .19, *p* < .10; see Supplement S5a).

In contrast, individuals with higher reasoning scores showed a more negative frontal FN amplitude in Block 1 following suspect faces (β_b1_ = −.20, *p* < .05; see Fig. 7b). These findings suggest that individuals with higher reasoning scores intensified the processing of correct feedback, especially following suspect faces in the initial task phase, and probably invested less in stimulus monitoring (cf. N2 data). In separate analyses for suspect versus nonsuspect faces, the reasoning effect for the frontal FN amplitude was neither significant for the latent slope and intercept frontal FN amplitudes nor for the measurement FN amplitude in Block 1 of mock suspect faces (see Supplement S4b) and mock nonsuspect faces (Supplement S5b).

The slope of the percentage of correct responses (i.e., the increase of correct responses across blocks) did not significantly differ across the three task blocks in individuals with higher versus lower reasoning (β_slope_= −.53, *p* = .19; see Fig. 7c). The same was true for the percentage of correct responses throughout the task (β_intercept_ = .27, *p* = .55; see Fig. 7c). The results of the Reasoning effect for the percentage of correct responses were also nonsignificant in separate analyses for mock suspect versus nonsuspect faces (see Supplement S4c and Supplement S5c).

### Hypothesis 4: Individuals with higher trait-BIS scores invest more conflict monitoring in the initial task block and show a more pronounced learning slope than individuals with lower trait-BIS scores

Individuals with higher trait-BIS scores revealed a more negative frontal N2 amplitude throughout the task (β_intercept_ = −.30, *p* < .05; see Fig. 7a) but not in the initial task block (β *=* .10, *p* = .16; see Fig. 7a). Separate analyses of the frontal N2 for mock suspect and nonsuspect faces revealed that the trait-BIS effect could be traced back to mock nonsuspect faces for the frontal N2 amplitude (β_intercept, separate_ = −.36, *p* < .01; see Supplement S4a). Individuals with higher versus lower trait-BIS scores did not significantly differ in their FN amplitudes following correct feedback (β_intercept_ = −.28, *p* = .12, β_slope_ = .57, *p* < .10; see Fig. 7b). Separate analyses for mock suspect and nonsuspect faces of the frontal FN showed no significant differences for trait-BIS scores in processing correct feedback to suspect faces more intensively in the initial task block (cf. beta weights of slope, intercept, and measurement Block 1; see Supplement S4b und S5b). A more pronounced learning effect for the percentage of correct responses was not observed for trait-BIS across task conditions (β_intercept_ = −.65, *p* = .25, β_slope_ = .20, *p* = .70; see Fig. 7c). Separate analyses for mock suspect and nonsuspect faces of the percentage of correct responses did not reveal any significant effects for trait-BIS and the percentage of correct responses following mock suspect faces (see Supplement S4c: β_intercept_ = −0.30, *p* = .40; β_slope_ = 0.16, *p* = .67) and nonsuspect faces (see Supplement S5c: β_intercept_ = −0.83, *p* = .23; β_slope_ = 0.38, *p* = .58) for individuals with higher trait-BIS scores.

### Hypothesis 5: A more pronounced FN component (i.e., more intense surprise of positive feedback) predicts a more pronounced (i.e., more positively motivated) learning slope of higher versus lower trait-BAS individuals

As indicated in Fig. 7a, individuals with higher versus lower trait-BAS scores did not significantly differ for the frontal N2 amplitude (β_intercept_ = .23, *p* = .08). These results were nonsignificant also for the frontal N2 amplitude with separate analyses on mock suspect and nonsuspect faces (see Supplement S5 and S5a). The frontal FN amplitude slope following correct feedback was significantly more negative for individuals with higher versus lower trait-BAS scores with time on task (β_slope_ = −.64, *p* < .01; see Fig. 7b) indicating that individuals with higher trait-BAS scores processed correct feedback more intensely with time on task. The intercept effect of the frontal FN amplitude revealed a less pronounced FN for higher versus lower trait-BAS individuals throughout the task (β_intercept_ = .37, *p* < .01; see Fig. 7b). In separate analyses of mock suspect versus nonsuspect faces, the frontal FN slope differed significantly for nonsuspect faces with higher trait-BAS scores coming along with a smaller (i.e., less positive) correct FN across task blocks (β_slope_ = −.67, *p* < .01; see Supplement S5b), but a more pronounced intercept frontal FN amplitude following nonsuspect faces (β_intercept_ = .33, *p* < .01; see Supplement S5b, no significant effects for mock suspect faces in Supplement S4b).

The percentage of correct responses was significantly higher for individuals with higher versus lower trait-BAS scores (β_intercept_ = .86, *p* < .05; see Fig. 7c). In separate analyses of mock suspect versus nonsuspect faces, the trait-BAS effect on the percentage of correct responses was neither observed following mock suspect faces (β_intercept_ = .13, β_slope_ = −.12; see Supplement S4c) nor following mock nonsuspect faces (β_intercept_ = .79, β_slope_ = −.73; see Supplement S5c).

## Discussion

The discussion of the present findings refers to our a priori hypotheses, and subsequent interpretations serve to describe the present findings in relation to previous studies. The present data suggest that more intense conflict monitoring across task blocks resulted in an increase of the overall percentage of correct responses indicating successful learning (see Fig. 6c; Hypothesis 1 was confirmed, but not for the prediction of the intercept-N2 on the slope percentage of correct responses; see Fig. 6b). That is, conflict monitoring was most intense (more negative N2) when participants just learned the correct stimulus classification (fewer %-correct responses). Moreover, the conflict monitoring intensity decreased with time on task followed by an increase of %-correct responses with time on task (see Fig. 6d). This finding does not confirm Hypothesis 1, but suggests that successful learning requires a higher degree of conflict monitoring at the beginning of the task. Observation by a socially relevant person intensified the conflict monitoring compared with nonobservation with time on task (Hypothesis 2 confirmed for slope, but not for intercept) and especially in the first task block to both types of faces. Individuals with higher versus lower reasoning scores processed correct feedback, especially in Block 1, following suspect faces more intensely, whereas conflict monitoring intensity to suspect and nonsuspect faces was reduced in Block 1. Thus, Hypothesis 3 was not confirmed for conflict monitoring, but we observed evidence for reasoning-related differences of feedback processing. Individuals with higher trait-BIS scores intensified their conflict monitoring throughout the task (Hypothesis 4 partly confirmed). Moreover, individuals with higher trait-BIS scores did not show a more pronounced FN amplitude following correct feedback throughout the task, and they did not significantly differ in learning measured by means of percentage of correct responses. The slope of the FN amplitude following correct feedback was more negative in individuals with higher trait-BAS versus lower trait-BAS scores with time on task (Hypothesis 5 not confirmed). This might be due to a less positive valence of our correct feedback with time on task for higher trait-BAS individuals. However, throughout the task (FN intercept; see Fig. 7b), the FN amplitude following correct responses was more pronounced (i.e., more positive FN amplitude) in individuals with higher trait-BAS scores. Moreover, in line with Hypothesis 5, the percentage of correct responses (see intercept, Fig. 7c) were higher for individuals with higher versus lower trait-BAS scores. According to rRST, higher trait-BAS is associated with higher sensitivity to reward. Therefore, the increased positivity of the intercept FN in higher trait-BAS individuals after correct feedback (which is probably rewarding) is compatible with the idea that a feedback-correct-related positivity co-occurs with the FN (Holroyd et al., 2008).

It is noteworthy, that individuals with higher reasoning scores revealed less intense conflict monitoring, but more intense feedback processing to predefined suspect faces in Block 1 to learn the correct stimulus classification as fast as possible (also at central cites of the FN component; see Supplement S2). Overall, our results show that individual differences of conflict monitoring and reinforcement learning as they have been obtained in (abstract) discrimination learning tasks (Scheuble et al., 2019) can also be obtained when the task is embedded into a forensic context. However, contextually nonembedded and embedded discrimination learning tasks evoke differential cognitive functioning (see also Nieden et al., 2020). Whereas higher reasoning scores came along with a more intense conflict monitoring (i.e., more negative N2 component) in Scheuble et al. (2019), we observed a less intense correct feedback processing in Block 1 (FN amplitude; see Fig. 7b) for individuals with higher reasoning scores. Moreover, the N2 amplitude in Block 1 was less intense in individuals with higher reasoning scores in the present task. Thus, individuals with higher reasoning scores processed the less frequently correct feedback to suspect faces in Block 1 more intensely (−.20), and this helped them to learn the correct stimulus–response relation. Thus, the forensic context modified the results predicted by the integrative conflict monitoring theory and reinforcement learning theory. Holroyd (2004) argued that the N2 and the FN component share the same time curve and topography so that both ERP components can be conceived to represent the same phenomenon. Our data reveal similar but not identical time curves of the N2 and FN, but both components suggest differential implications for conflict monitoring and cognitive control.

Individuals with higher trait-BIS scores primarily invested more conflict monitoring for successful trial-and-error learning than lower trait-BIS individuals. Higher trait-BIS individuals applied conflict monitoring (i.e., stimulus differentiation) more proactively in advance of the external feedback to learn the stimulus classification by trial and error in the initial task block. Thus, our data provide evidence for a proactive mechanism of control that facilitates learning in individuals with higher trait-BIS scores (e.g., Botvinick & Braver, 2015; Leue et al., 2014). Individuals with higher reasoning scores and higher trait-BAS scores reactively activated their feedback processing in order to facilitate learning in a trial-and-error discrimination learning task. These findings suggest a more feedback-focused neural process during trial-and-error learning in higher reasoning and trait-BAS individuals, especially in the initial learning phase, as the stimuli probably do not yield sufficient information for stimulus classification based on conflict monitoring. Higher trait-BIS individuals revealed evidence for intensified conflict monitoring during trial-and-error learning. This finding is in line with previous results on trait-BIS and conflict monitoring obtained in go/no-go tasks without a mock forensic context (Amodio et al., 2008; Leue et al., 2012). Moreover, Moser, Moran, Schroder, Donnellan, and Yeung (2013) reported that an enhanced error-related negativity (ERN/Ne) in more-anxious individuals yields less efficient error monitoring because more resources for error monitoring are required to successfully perform a task. Our data correspond to the argument of Moser et al. (2013) that anxious individuals invest more cognitive resources to learn the stimulus–response relation successfully. As reported in Moser et al. (2013), more-anxious versus less-anxious individuals did not differ in observable task performance. As participants were not instructed in how to best learn the correct stimulus classification (suspect vs. nonsuspect), we conclude that more-anxious individuals invested higher conflict monitoring intensity proactively to prevent errors (cf. for a similar argumentation, see Leue, Weber, Elger, Trautner, & Beauducel, 2018). Cavanagh and Shackman (2015) also highlight in a meta-analysis that the frontal-midline theta signal, which reflects activity of the midcingulate cortex, is modulated by anxiety and predicts behavioral adaptation. Thus, professionals in a forensic context (e.g., police officers, lawyers), with higher anxiety scores, are more likely to apply intensified conflict monitoring (i.e., use the most initial information available) to process all or most relevant information correctly. In contrast, for individuals with higher reasoning and trait-BAS scores, respectively, the most initial stimulus information seems to be less informative, so that they focus their information processing on feedback to appropriately learn and adapt behavior (e.g., response type, decisions).

Moreover, our data reveal that stimuli that should be learned by trial and error do not evoke an internal error signal until the stimulus–response association has been successfully learned (cf. first indicator hypothesis). The first indicator hypothesis predicts that errors performed very soon after a response interval do not provide an internal error signal that could be measured by the ERN/Ne, whereas errors that are made or feedbacked later after a response interval evoke an external error signal that can be measured by means of FN (Stahl, 2010). Therefore, we conclude that the more pronounced FN amplitude following correct feedback (see Supplement S3) served as an external signal that facilitated learning in our task (Holroyd, Krigolson, Baker, Lee, & Gibson, 2009; Stahl, 2010). Future research is needed to see whether our preliminary explanation of the results on the correct FN and learning holds as an extension of the first-indicator hypothesis. The learning curves in our study were similar to the learning curves presented in Bellebaum et al. (2010) for the three task blocks, and as reported in Sailer, Fischmeister, and Bauer (2010) for the individual learning curves of high learners.

Our data provide evidence on the monitoring processes and on the behavioral adaptation during trial-and-error learning based on figural stimuli (faces). Thus, results that have been obtained for trait-BIS, trait-BAS, and reasoning in conventional go/no-go tasks and discrimination learning tasks could also be obtained when the task was embedded in a mock forensic context. Moreover, when police officers and lawyers integrate information to reconstruct an offense, they include an even considerably higher number of information and different types of information (e.g., verbal, figural, numerical). Therefore, future studies might aim at replicating our findings and compare verbal, figural, and numerical monitoring processes during trial-and-error learning in a forensic context. Moreover, our analyses focus on conflict monitoring (N2 component) and feedback processing (FN component) of correctly performed trials, while leaving analysis of ERN/Ne aside because of too few incorrect responses per task block. However, other learning studies investigated performance monitoring by means of ERN/Ne (Thoma & Bellebaum, 2013). Participants demonstrated a strong learning curve, providing evidence of high motivation to perform the task and high compliance with task instructions. Moreover, the strong increase of the learning slope, especially in Block 1, resulted in a robust and good performance in the second and the third task blocks (for motivational effects and FN, see Yeung, Holroyd, & Cohen, 2005). Therefore, an analysis of the ERN/Ne would have suffered from fewer participants with a reliable number of artifact-free ERN/Ne epochs (for reliability results, see Meyer, Riesel, & Hajcak Proudfit, 2013; Olvet & Hajcak, 2009). Finally, future research might clarify the type of trend (e.g., quadratic, nonlinear) underlying the processing of correct feedback in higher trait-BIS individuals. The slope for the correct FN revealed that higher trait-BIS individuals did not yield a linear trend of processing correct feedback intensity. In this line, future research might elucidate whether the differential effects reported in this study hold for long-term feedback–behavior relations introduced in hierarchical reinforcement learning (Osinsky, Ulrich, Feser, Gunawardena, & Hewig, 2017). Similarly, the relevance of probabilistic feedback in comparison to deterministic feedback for individual differences of reinforcement learning should be probed (e.g., Bakic, De Raedt, Jepma, & Pourtois, 2015; Eppinger, Kray, Mock, & Mecklinger, 2008; Rustemeier, Schwabe, & Bellebaum, 2013). Moreover, future research should investigate the generalizability of the present BIS/BAS data by means of more recent German psychometric BIS/BAS measurements (Leue, 2015; Pugnaghi et al., 2017; Reuter et al., 2015).

### Limitations and future directions

In the present study, we did not control for a diminution of the N2 amplitude by means of the alpha band, which has been observed for auditory stimulus material (Barry et al., 2004). Thus, another study could control for the effect of alpha activity on the N2 amplitude in visual stimulus material. In accordance with studies investigating effects of the high-pass filter on ERP magnitudes (Tanner, Morgan-Short, & Luck, 2015; Widmann et al., 2015), we aim at applying the same 1 Hz high-pass filter for N2 analyses and FN analyses. N2 processes should also be controlled for motor effects (e.g., correct vs. incorrect execution of a correct motor plan) and functional network effects as indicated for ERN and FN data (Hewig, Coles, Trippe, Hecht, & Miltner, 2011). Finally, we used a go/no-go discrimination learning task with an equal probability of go and no-go stimuli. Thus, we acknowledge that the results obtained in the present study may not be comparable with the results of go/no-go tasks using an equal or unequal probability of go and no-go stimuli without learning (e.g., Larson et al., 2014, p. 290).

Moreover, we denoted our task as a discrimination learning task, although such tasks are sometimes labeled as equal probability go/no-go tasks (Barry & De Blasio, 2013; Barry, De Blasio, & Cave, 2016). Although our results provide further evidence for the N2 component in a discrimination learning task with equal probability go/no-go stimuli, this line of research should be expanded to further our understanding of the functional implications of the N2 components in such tasks. To disentangle response preparation and pure stimulus comparison, a principal component analysis might be performed in another study to decompose frontal stimulus-locked N2 components from other N2/P3 components that might indicate response preparation. Finally, future research might address the generalizability of N2 and frontal P3 processes of monitoring and feedback learning in a discrimination learning task (cf. Folstein & van Pettern, 2008).

### Conclusion

Our study provides findings supporting predictions on individual differences of conflict monitoring, feedback processing, and reinforcement learning in a mock forensic context (i.e., a semantically embedded discrimination learning task). We obtained new insights on primary cognitive processes applied during reinforcement learning and individual differences. Social observation intensified conflict monitoring throughout the learning task, suggesting that social observation is another context variable beyond cognitive demand and reinforcement that facilitates conflict monitoring. Individuals with higher reasoning ability and individuals with higher trait-BAS scores processed the feedback more intensely, whereas individuals with higher anxiety scores monitored the faces more intensively to learn the correct face classification. Our data in a mock forensic task setting reveal comparable learning curves as reported in other learning tasks, and therefore support prior findings that the strongest learning increase occurs in initial learning phases. We conclude that more-anxious individuals proactively invest more conflict monitoring to learn successfully, whereas individuals with higher reasoning scores, especially in the first task block, and individuals with higher trait-BAS scores throughout the task reactively process feedback more intensively during learning.

## Electronic supplementary material

ESM 1 (DOCX 1.29 mb)
