# Supplementary material for: Individual differences of conflict monitoring and feedback processing during reinforcement learning in a mock forensic context
Source: Cogn Affect Behav Neurosci. 2020 Feb 10;20(2):408–26. doi: 10.3758/s13415-020-00776-7 (PMC7105439; doi:10.3758/s13415-020-00776-7)
Supplement: Supplementary file 1 — (DOCX 1.29 mb) [file 13415_2020_776_MOESM1_ESM.docx]

Supplement S1:

Case 277/1314507: Murder case in Klaus-Mann-Str. 277. Background: Mr. Axel Thiel (subsequently Mr. T.) from Münster owns several buildings, one of them is located in Klaus-Mann-Str. 277. It is an older building with currently 12 renters. A few months ago, Mr. T. received an extra-ordinary good offer from a potential buyer. The potential buyer wants to tear down the building and to re-build expensive luxury apartments. On April 1^st^, Mr. T. announces the sale on a renter assembly. The renters are also informed about the demolition of the building. One hour after the assembly, Mr. T. is found dead in the staircase. Previous inquiries: Cause of death was a broken neck due to a downfall. The coroner Dr. Apfel eliminated an accident without involvement of other people as a possible cause of death due to the position Mr. T. was found in. Small pieces of clothes were found on the clothes of the victim and confirm that Mr. T. was thrown to death on the steep stairs. Dr. Apfel states that one wouldn’t need much strength for it, it only needed a light kick. This means, the perpetrator could either be male or female, young or old. Because of the previous inquiries, the circle of suspects could be narrowed down to the renters of the building Klaus-Mann-Str. 277: The personal assistant of Mr. T. stated that only the renters, Mr. T., herself and the caretaker Mr. Prahl were present in the building. (The assistant and Mr. Prahl have a credible alibi and are not suspected.) Mr. Prahl declared that every renter had a private conversation with Mr. T. after the assembly. Furthermore, the forensic investigation indicated that the cloth pieces that were found on the jacket of the victim must come from one of the apartments of the building. Possible motive: All renters have been lived in the building for several years. The sale and the demolition would have forced everyone to shortly move and to leave a loved environment. The sole heiress of Mr. T. is his daughter, Ms. Tanja Thiel. Ms. Thiel stated that she won’t sell the building because of childhood memories. Ms. T. was in touch with the renters for a long time, because of several administrative activities. Thus, every renter knew that she would not sell the building.

Supplement S2: Growth model of the central N2 amplitude (A), central FN amplitude (B) and percentage of correct responses (C) for mock suspect and non-suspect faces across three task blocks. ** *p* < .01; * *p* < .05; (*) *p* < .10; ns = non-significant. All *p*s are reported two-tailed.


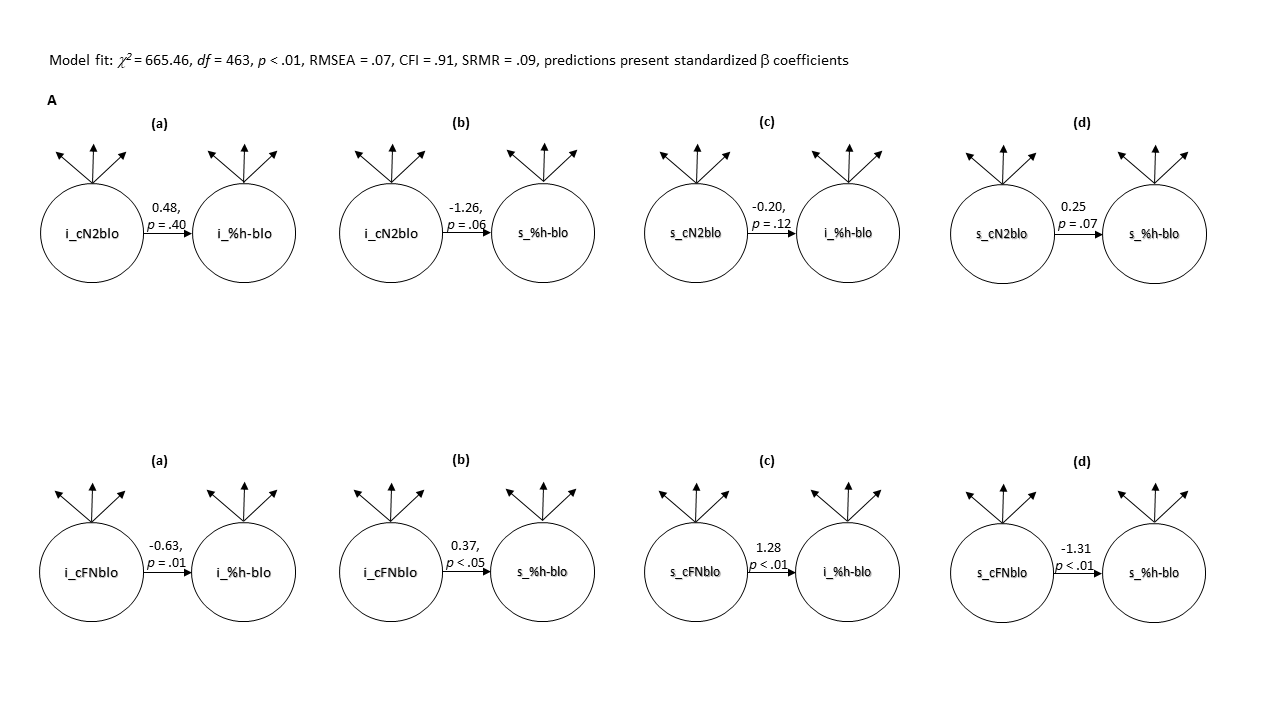


The predictions for the central N2 data were not significant. A more intense overall central FN amplitude predicted a higher overall percentage of correct responses (*β* = -.63). A significantly more negative central FN slope across task blocks was related to a significantly higher increase of the percentage of correct responses (*β* = -1.31). The relationship between intercept and slope FN amplitudes with the respective slope and intercept percentage of correct responses resulted in significant positive beta-coefficients (b) and (c) meaning that less negative central FN amplitudes (intercept and slope) came along with higher percentages of correct responses (slope and intercept).


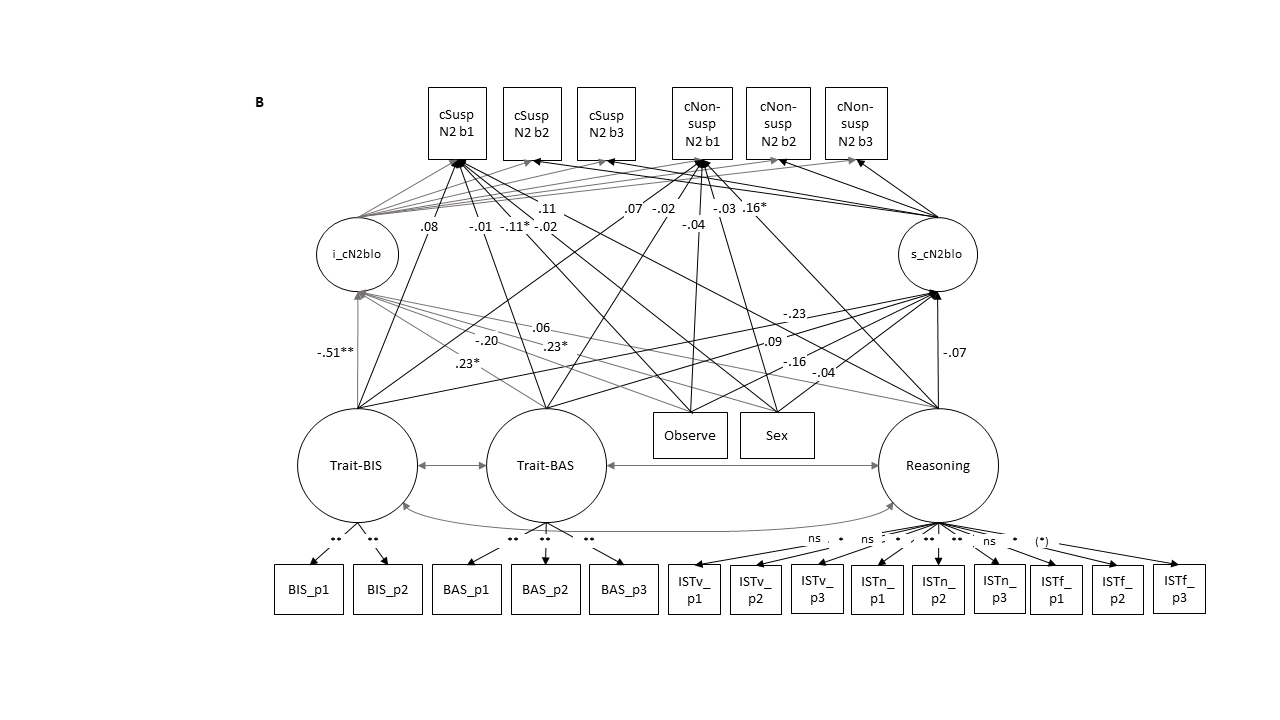


Results for the central N2 amplitude were widely similar to the results for the frontal N2 amplitude with the following exceptions: Observation was significantly related to a more negative central N2 amplitude. Individuals with higher Trait-BAS scores showed a less negative central N2 amplitude (intercept). Observation was not significantly related to the central N2 amplitude following mock non-suspect faces. The path coefficient for observation and the central N2 slope was not significant.


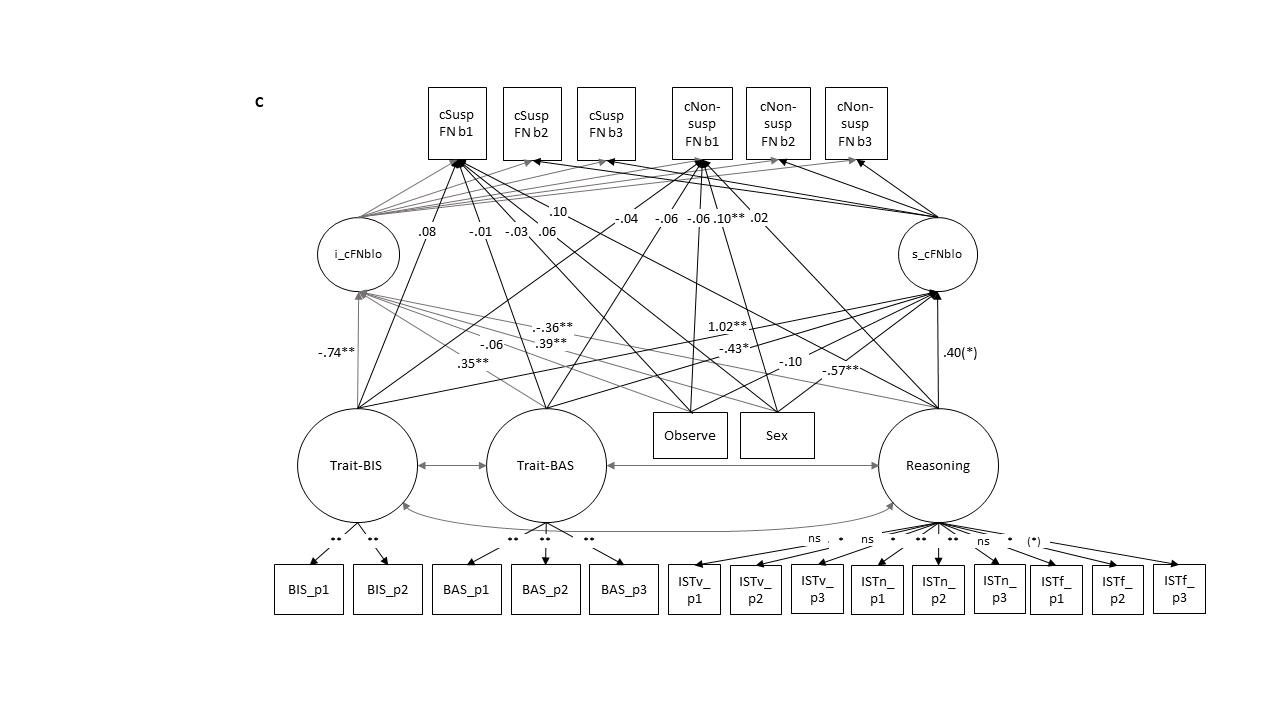


The results of the frontal FN amplitude were widely similar to the results for the central FN amplitude. Four further path coefficients were significant for the central FN amplitude compared to the frontal FN amplitude. Individuals with higher trait-BIS scores and individuals with higher reasoning scores showed a more negative overall central FN amplitude (intercept). The slope of the central FN amplitude became less negative across task blocks for individuals with higher trait-BIS scores. Women showed a more negative central FN slope compared to men.


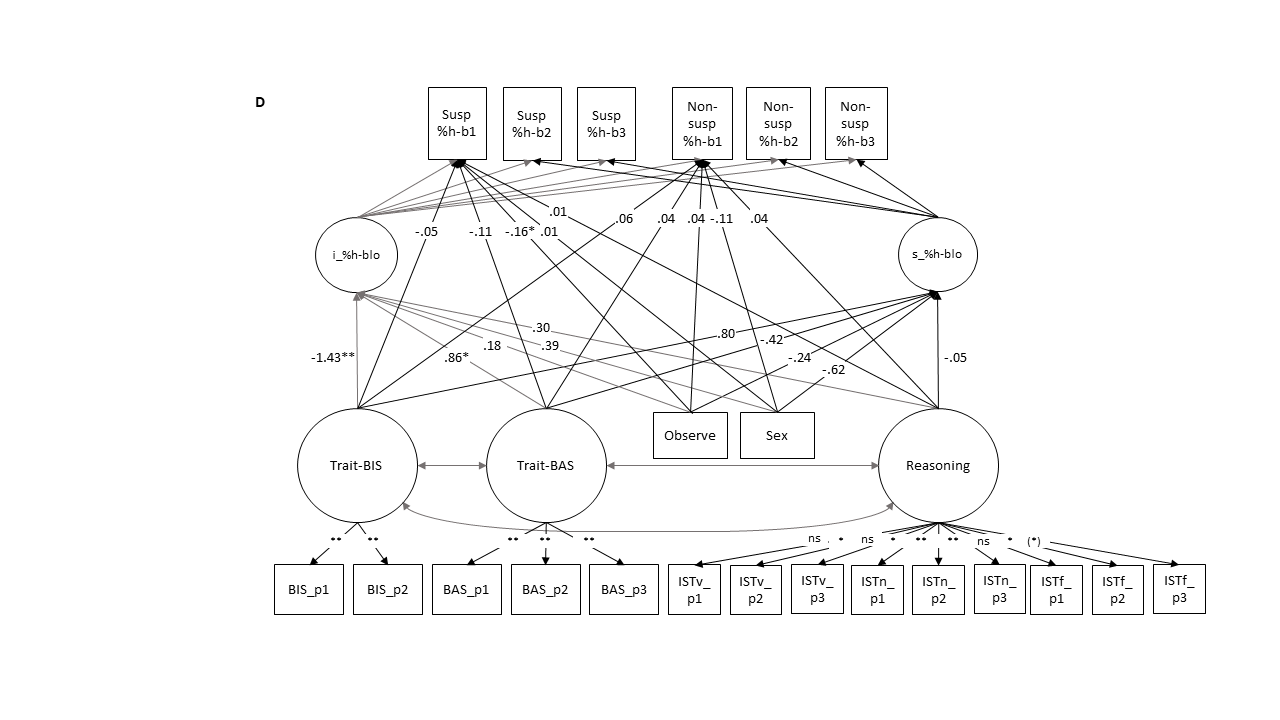


Results for the percentage of correct responses were identical with the results in the model with frontal N2 and frontal FN amplitudes with one exception and non-significant with two exceptions: The path coefficient for trait-BIS and the intercept of the percentage of correct responses (i_%h-blo) was significant indicating that individuals with higher trait-BIS scores revealed a lower percentage of correct responses (*β_intercept_* = -1.43).when the complete growth model included the central N2 and central FN amplitudes. Moreover, the percentage of correct responses to mock suspect faces in block 1 was higher for participants who had been observed while they performed the experimental task (β = -.16).

The growth model for central N2, central FN amplitudes and percentage of correct responses revealed the following correlations between trait-BIS, trait-BAS, Reasoning, observation, and sex: Trait-BAS and trait-BIS correlated *r* = -.01. Trait-BAS and reasoning correlated *r* = .22, *p* < .05, trait-BSA and sex, *r* = -.02, and trait-BAS with observation, *r* = -.10. Trait-BIS and reasoning correlated r = -.44, *p* < .01, trait-BIS and sex, *r* = .65, *p* < .01, and trait-BIS with observation, *r* = -.12. Reasoning and sex correlated r = -.32, p < .01, and Reasoning with observation, *r* = .08.

Supplement S3: frontal FN and learning predictions (i = intercept, s = slope; %h-blo = percentage of correct responses across task blocks, ** *p* < .01). All *p*s are reported two-tailed.


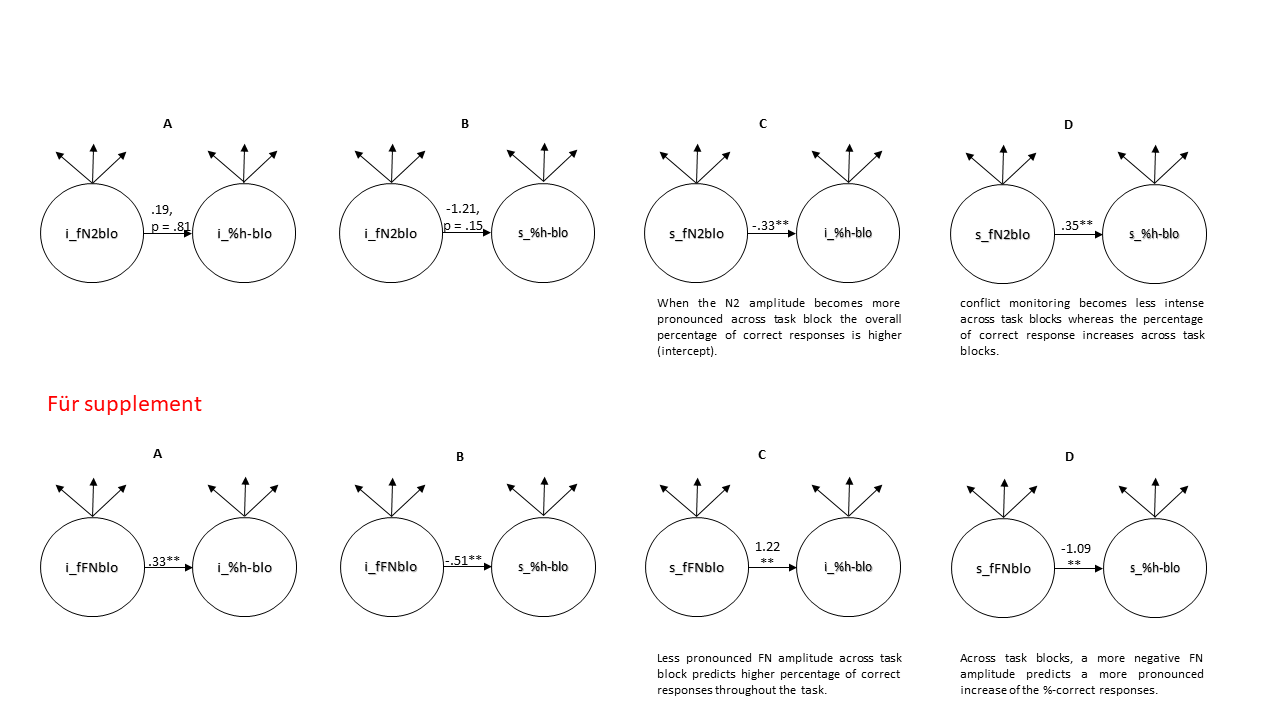


Supplement S4: Growth model of the frontal N2 amplitude (A), frontal FN amplitude (B) and percentage of correct responses (C) for mock suspect faces across three task blocks. ** *p* < .01; * *p* < .05; (*) *p* < .10. ns = non-significant. All *p*s are reported two-tailed.
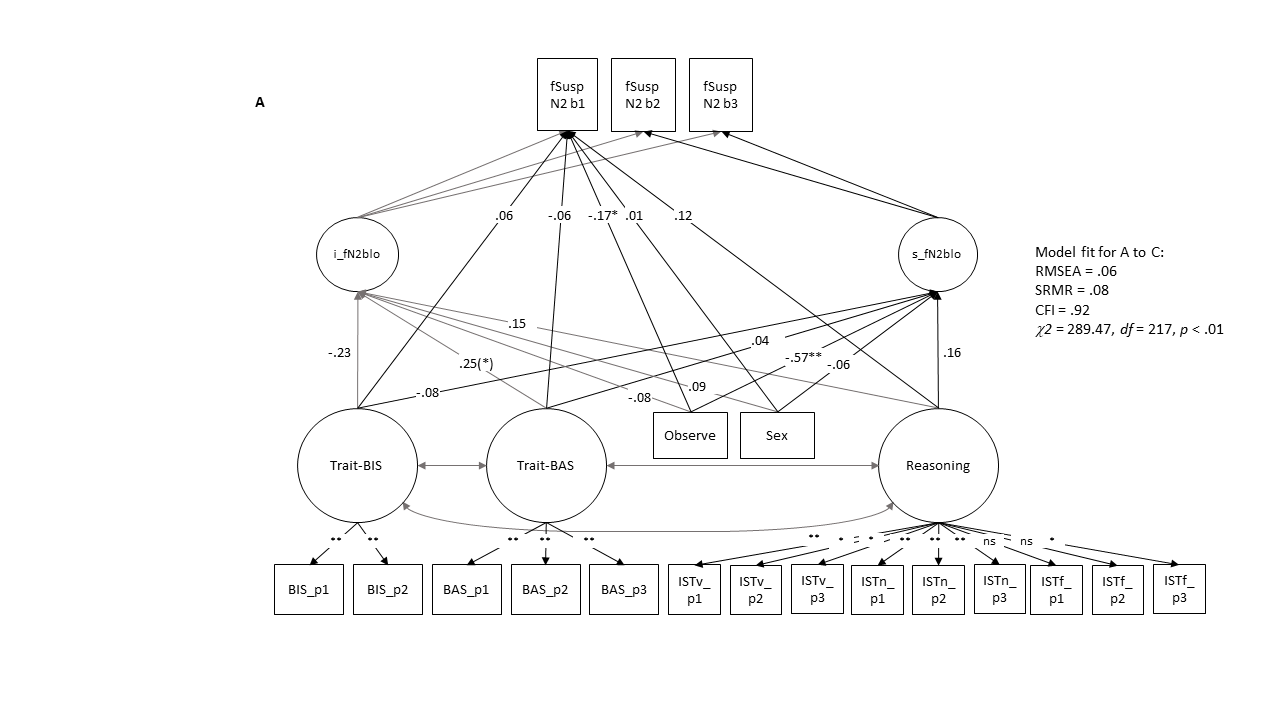


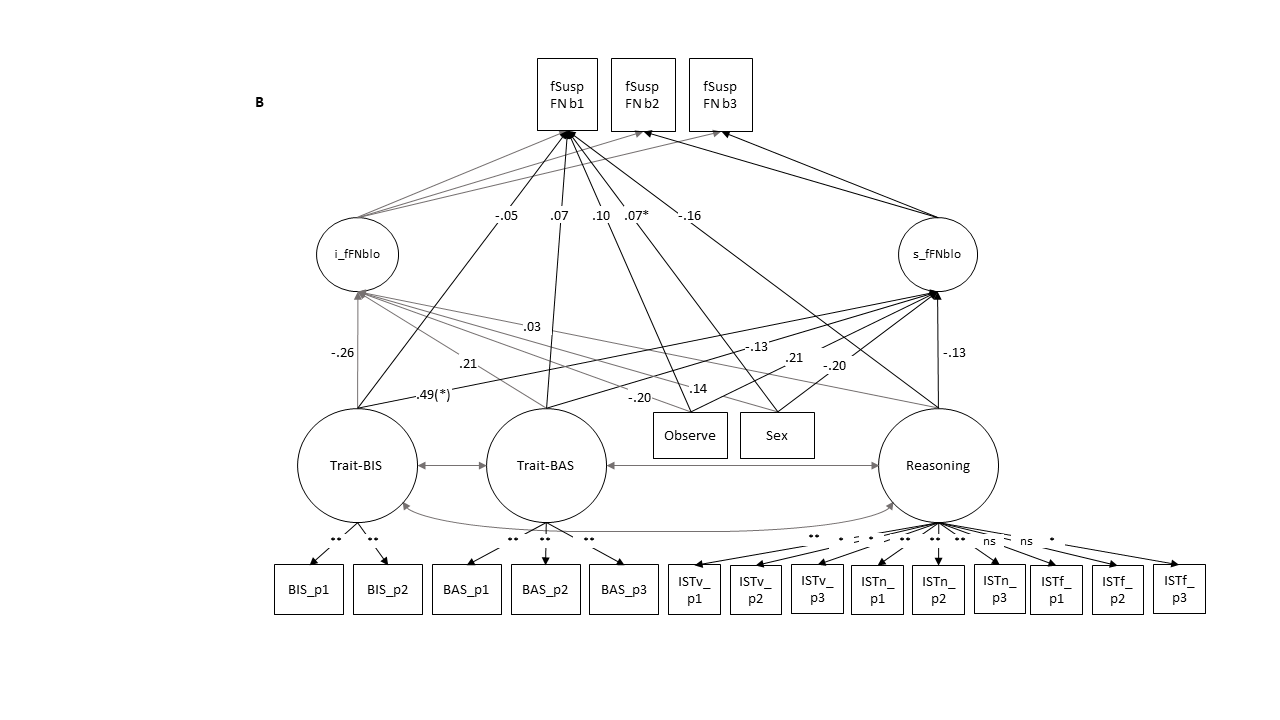


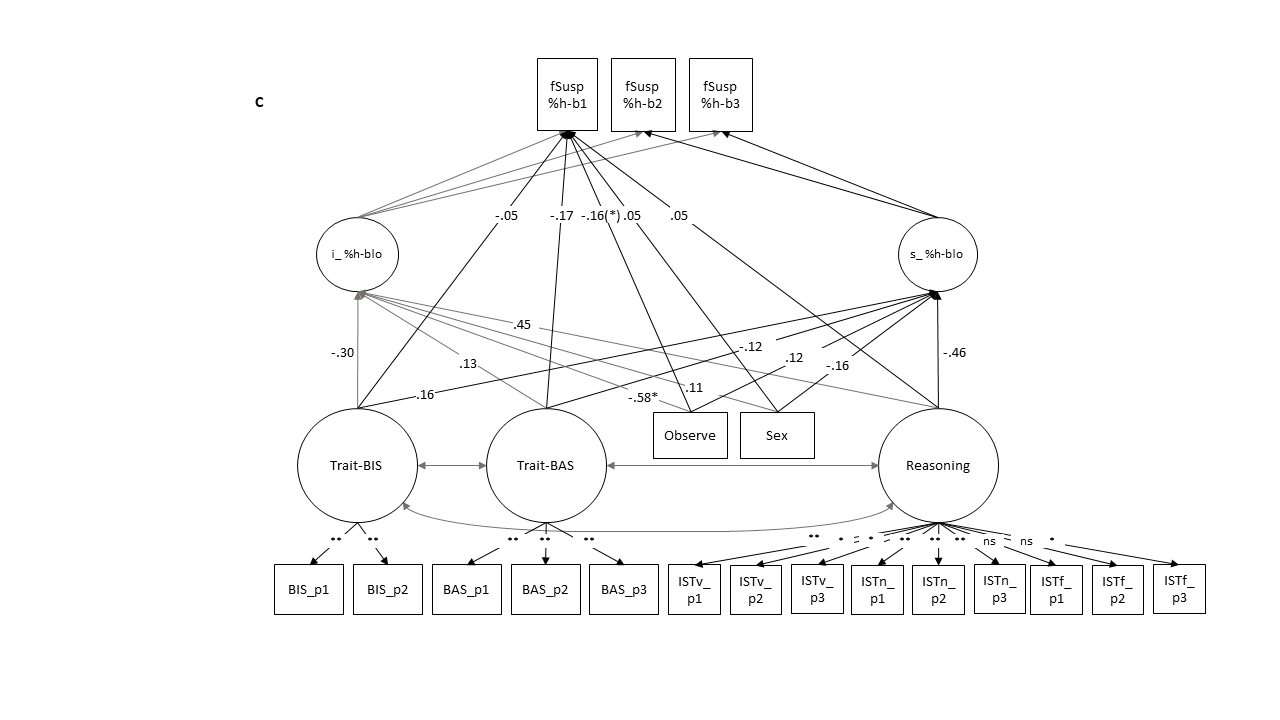


Supplement S5: Growth model of the frontal N2 amplitude (A), frontal FN amplitude (B) and percentage of correct responses (C) for mock non-suspect faces across three task blocks. ** *p* < .01; * *p* < .05; (*) *p* < .10. ns = non-significant. All *p*s are reported two-tailed.


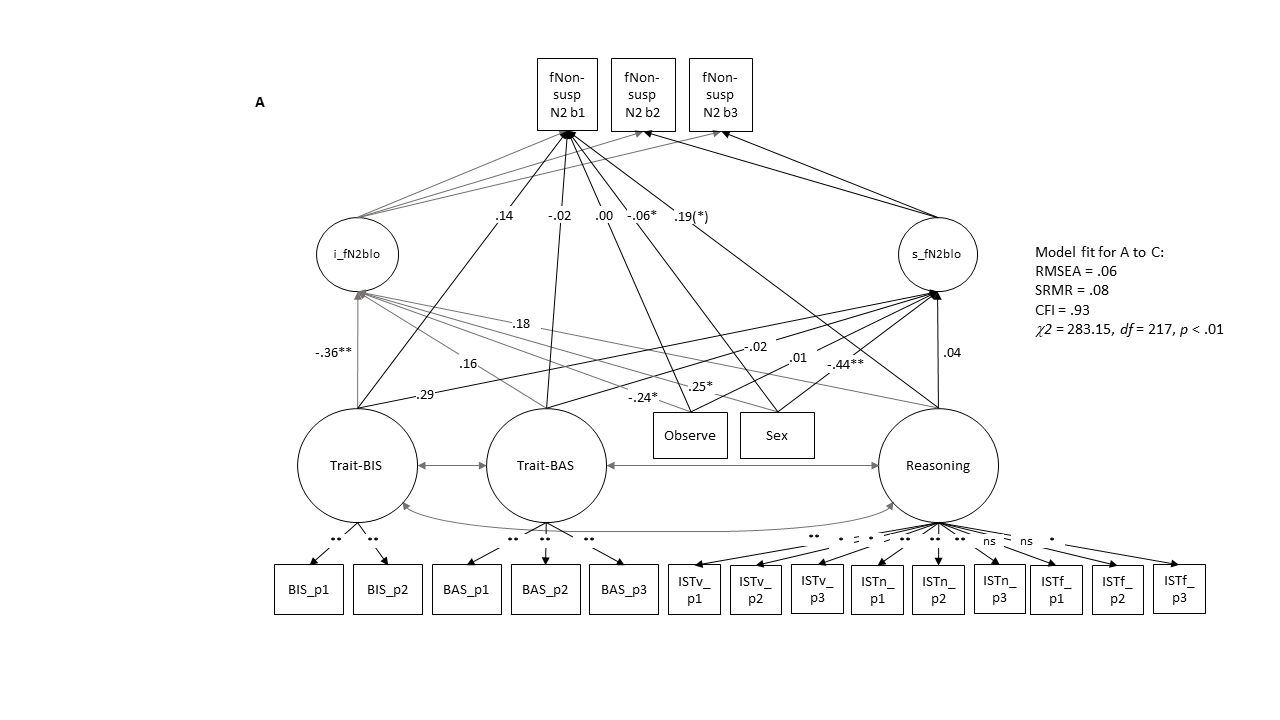


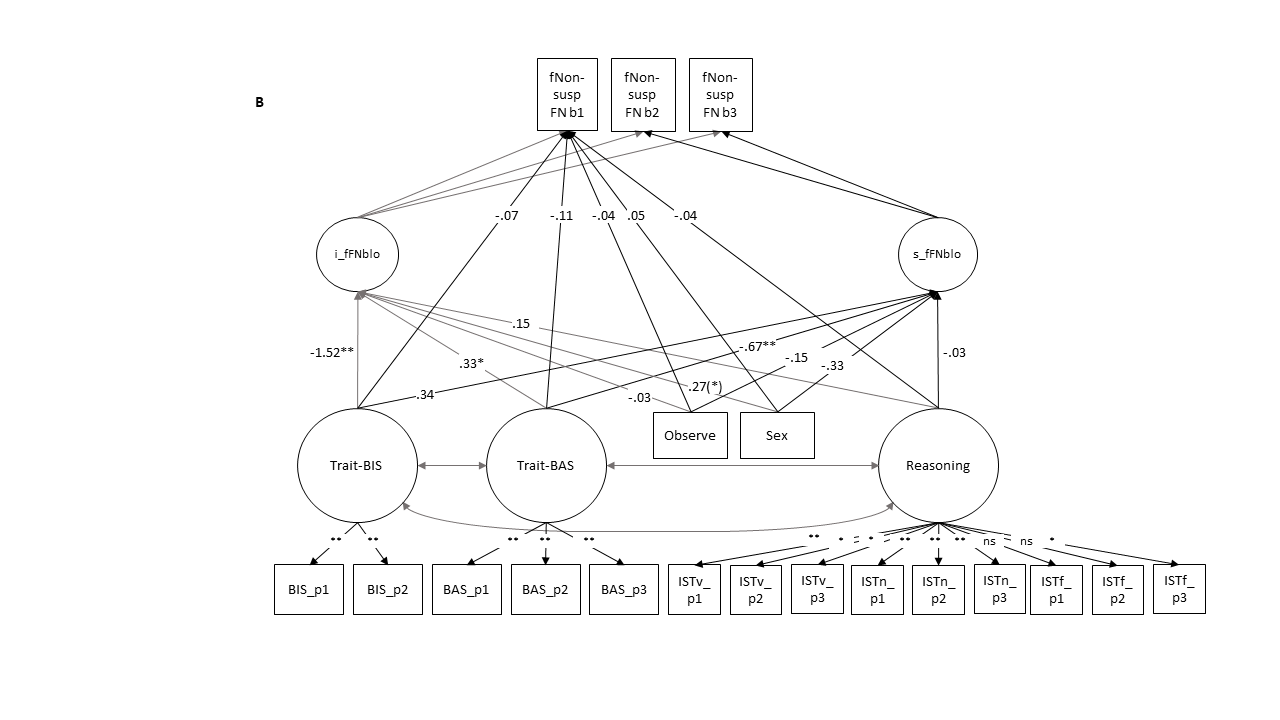


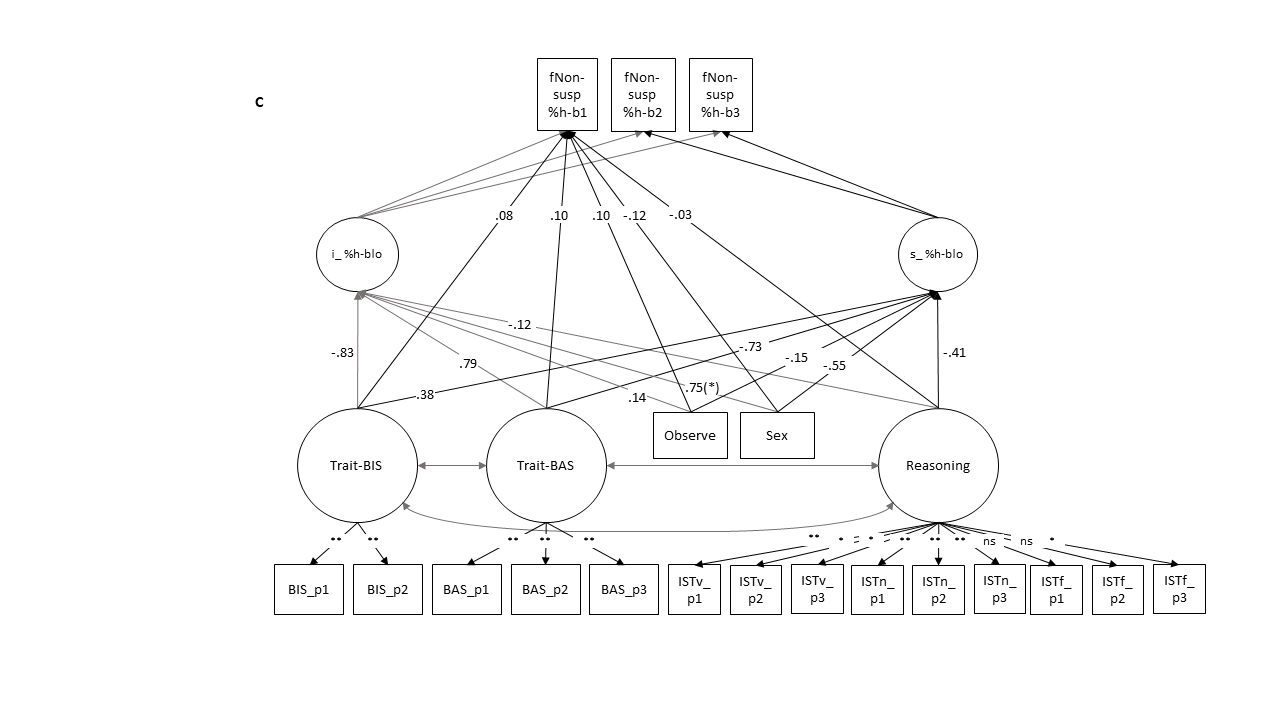


Supplement S6 for results section: Post-hoc analyses of the overall growth model

Mock s*uspect vs. non-suspect main effect on FN*

The effect of the within-subjects factor mock suspect vs. non-suspect faces on FN amplitudes was not part of the growth model comprising all dependent variables because the number of free parameters would have been too large so that the model would not have been identified. Since the effect of mock suspect vs. non-suspect faces on FN amplitudes is nevertheless interesting, we calculated two additional growth models that were only based on the FN amplitudes as dependent variables. Therefore, the degrees of freedom of the additional models are smaller than for the overall model: One where the intercepts of the FN amplitudes were allowed to vary across mock suspects and non-suspects (*χ^2^* = 7.77, *df* = 9, *p* = .558, RMSEA = .00, SRMR = .04, CFI = 1.00) and a second model where equality constraints were imposed for the FN amplitudes of mock suspects and non-suspects (*χ^2^* = 18.71, *df* = 12, *p* < .096, RMSEA = .08, SRMR = .05, CFI = .98). The corresponding test for the Satorra-Bentler scaled *χ^2^* difference was significant (*χ^2^* = 13.70, *df* = 3, *p* < .01) revealing that the FN for mock suspect faces was more negative (*M* = -2.91, *SE* = 0.60) than the FN for mock non-suspect faces (*M* = -2.05, *SE* = 0.60).

*The trait-BIS × observation interaction-term*

Since higher trait-BIS scores were associated with more negative frontal N2 amplitudes throughout the task and since observation intensified conflict monitoring across task blocks, we performed a post-hoc investigation of the trait-BIS × observation interaction-term as a predictor of the (frontal) N2 amplitudes and the FN amplitudes in the first task block and as a predictor of the intercepts and slopes of the N2 and FN. In order to limit the number of free parameters and in order to use the Satorra-Bentler scaled robust maximum likelihood estimator, this additional model was based on scales (measured variables) for trait-BIS and trait-BAS, the observation variable, and the trait-BIS × observation interaction-term as independent variables and the respective measured variables, intercepts, and slopes for the N2 and the FN as dependent variables. The model fit was excellent (*χ^2^* = 83.03, *df* = 79, *p* = .35, RMSEA = .02, SRMR = .04, CFI = 1.00). The trait-BIS × observation interaction was a significant positive predictor of the N2 intercept (*β_intercept_* = .31, *p* < .01) and a significant negative predictor of the N2 for mock suspects in the first block (*β_b1_* = -.13, *p* < .05). The significant negative path coefficient reveals that higher trait-BIS individuals showed more negative N2 amplitudes for mock suspects in the first block when they were observed by significant others. In contrast, the significant positive path coefficient reveals that higher trait-BIS individuals showed less negative N2 amplitudes across all blocks when observed by significant others.

The trait-BIS × observation interaction was not a significant positive predictor of the FN for mock suspects in the first block (*β_b1_* = .11, *p* = .08) but a significant positive predictor of the FN for mock non-suspects in the first block (*β_b1_* = .14, *p* < .05). Thus, higher trait-BIS individuals show slightly smaller FN amplitudes in the first block when observed by significant others.

Discussion of the post-hoc analyses:

Post-hoc analyses revealed there was a main effect for mock suspect versus non-suspect faces with more negative FN amplitudes for suspect faces. This indicates that feedback on mock suspect faces was more intensely processed. There could be two reasons for this effect: It could be that the feedback was more intensely processed because mock suspect faces were go stimuli. It is, however, also possible that the feedback on mock suspect faces was more intensely processed because suspect faces were more relevant according to the mock forensic vignette.

As becomes apparent from post-hoc analysis, the effect of higher trait-BIS on conflict monitoring was also modulated by observation of significant others. Interestingly, conflict monitoring was more enhanced for mock suspects in the first block of the task when higher trait-BIS individuals were observed by significant others. Overall, conflict monitoring across the whole task was reduced when higher trait-BIS individuals were observed by significant others suggesting that a disengagement from a higher degree of conflict monitoring follows when the person x situation constellation becomes too intense (ceiling effect). The FN in the first block was also reduced in higher trait-BIS individuals when they were observed by significant others. The results of the observation condition in higher trait-BIS individuals indicate two effects: On the one hand, they detract overall attention leading to reduced conflict monitoring and smaller FN amplitudes. On the other hand, they lead to an enhanced conflict monitoring for mock suspects. Perhaps, higher trait-BIS individuals compensate for overall detraction of attention when observed by significant others by means of an enhanced focus on mock suspects. As these results were based on a post-hoc analysis, further research is needed to see whether these findings could be replicated.
